# Solar-Driven Thermochemical Splitting of CO_2_ and *In Situ* Separation of CO and O_2_ across a Ceria Redox Membrane Reactor

**DOI:** 10.1016/j.joule.2017.07.015

**Published:** 2017-09-06

**Authors:** Maria Tou, Ronald Michalsky, Aldo Steinfeld

**Affiliations:** 1 Department of Mechanical and Process Engineering, ETH Zürich, 8092 Zürich, Switzerland

## Abstract

Splitting CO_2_ with a thermochemical redox cycle utilizes the entire solar spectrum and provides a favorable path to the synthesis of solar fuels at high rates and efficiencies. However, the temperature/pressure swing commonly applied between reduction and oxidation steps incurs irreversible energy losses and severe material stresses. Here, we experimentally demonstrate for the first time the single-step continuous splitting of CO_2_ into separate streams of CO and O_2_ under steady-state isothermal/isobaric conditions. This is accomplished using a solar-driven ceria membrane reactor conducting oxygen ions, electrons, and vacancies induced by the oxygen chemical potential gradient. Guided by the limitations imposed by thermodynamic equilibrium of CO_2_ thermolysis, we operated the solar reactor at 1,600°C, 3·10^−6^ bar $p_{{\text{O}}_{2}}$ and 3,500 suns radiation, yielding total selectivity of CO_2_ to CO + ½O_2_ with a conversion rate of 0.024 μmol·s^−1^ per cm^2^ membrane. The dynamics of the oxygen vacancy exchange, tracked by GC and XPS, further validated stable fuel production.

## Highlights

• Concentrated solar energy drives the thermochemical splitting of CO_2_ to CO and O_2_
• Single-step continuous process is performed across a redox ceria membrane
• Solar reactor is operated under steady-state isothermal/isobaric stable conditions
• Total selectivity and *in situ* separation is obtained for products CO and O_2_

## Context & Scale

Solar technologies that facilitate the efficient conversion of CO_2_ into fuels offer a sustainable path to the production of renewable drop-in hydrocarbons for the transportation sector, especially for aviation, without the need to replace the existing massive global infrastructure for their storage, distribution, and consumption. We pursue the direct use of concentrated solar radiation as the source of high-temperature process heat to thermochemically split CO_2_ into separate streams of CO and O_2_. This is accomplished via a single-step continuous process across a redox membrane using a compact solar reactor design. The stable steady-state experimental results obtained with total selectivity under realistic high-flux operational conditions, along with the potential for high solar-to-fuel energy conversion efficiencies, support the viability of the modular membrane reactor technology for converting CO_2_ to fuels at a large scale by integration with the established concentrating solar tower and dish systems.

## Graphical Abstract

**Figure:**
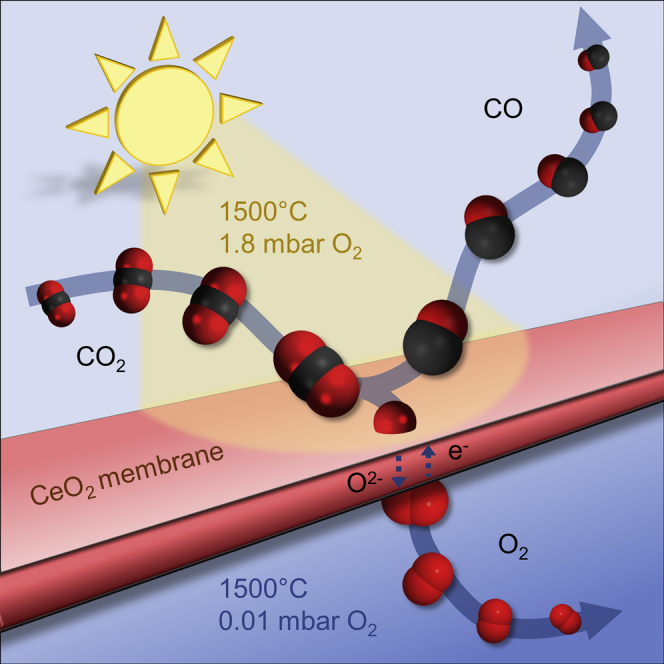

## Teaser

Conversion of CO_2_ into fuels via a solar-driven thermochemical process enables the efficient production of sustainable transportation fuels. The solar reactor continuously splits CO_2_ across a redox membrane into separate streams of CO and O_2_ using concentrated solar radiation. The stable experimental results and the theoretical solar-to-fuel energy conversion efficiency support the viability of the modular solar reactor technology for converting CO_2_ to fuels at a large scale by integration with the established concentrating solar tower and dish systems.

## Introduction

Developing solar technologies for converting CO_2_ into fuels has become a grand energy challenge, as it closes the anthropogenic carbon cycle and leads to the production of sustainable transportation fuels.^1^, ^2^ The thermochemical approach is particularly appealing because the entire solar spectrum is utilized in the form of high-temperature process heat to drive a cycle based on metal oxide redox reactions.^3^ Nonstoichiometric ceria $\left({\text{CeO}}_{2\text{-}\text{δ}}\right)$ has emerged as an attractive redox active material because of its crystallographic stability and fast oxygen ion diffusivity.^4^, ^5^, ^6^ The two-step redox cycle is represented by:

(Equation 1) $${\text{CeO}}_{{2\text{-}\text{δ}}_{\text{ox}}}\to {\text{CeO}}_{{2\text{-}\text{δ}}_{\text{red}}}+\frac{\Delta \text{δ}}{2}{\text{O}}_{2}$$

(Equation 2) $${\text{CeO}}_{{2\text{-}\text{δ}}_{\text{red}}}+\Delta \text{δ}\;{\text{CO}}_{2}\to {\text{CeO}}_{{2\text{-}\text{δ}}_{\text{ox}}}+\Delta \text{δ}\;\text{CO}$$

In the endothermic reduction step driven by concentrated solar energy, Equation 1, ${\text{CeO}}_{{2\text{-}\text{δ}}_{\text{ox}}}$ is reduced to ${\text{CeO}}_{{2\text{-}\text{δ}}_{\text{red}}}$ and O_2_ is liberated. The total number of oxygen vacancies formed in the ceria crystal lattice sets the upper limit for the specific oxygen exchange capacity, given by Δδ = δ_red_ – δ_ox_. In the exothermic oxidation step, Equation 2, ${\text{CeO}}_{{2\text{-}\text{δ}}_{\text{red}}}$ is re-oxidized by CO_2_ to generate CO. In principle, the redox cycle can be operated under a temperature/pressure-swing mode to control Δδ and thereby the fuel yield per cycle,^7^ but these swings induce significant material stresses and energy irreversibilities.^8^, ^9^, ^10^, ^11^ Both temperature and pressure swings can be completely eliminated by means of a ceria membrane reactor that establishes a spatial separation between the reduction and oxidation sites and conducts oxygen ions, electrons, and vacancies driven by the oxygen chemical potential gradient across the membrane. With such an arrangement, both steps of the redox cycle can be performed simultaneously and continuously at isothermal/isobaric steady-state conditions. This in turn enables the design of a compacter solar reactor. Pioneering studies on solar thermal membrane reactors for splitting H_2_O were conducted by Fletcher and co-workers.^12^, ^13^ Previous experimental studies with membrane reactors were non-solar and made use of a sacrificial reducing agent, such as CH_4_,^14^, ^15^, ^16^, ^17^ CO,^18^, ^19^ or H_2_^20^, ^21^ to scavenge the oxygen produced, thus failing to accomplish the net splitting of CO_2_, i.e., CO_2_ → CO + ½ O_2_. Here, we experimentally demonstrate for the first time the splitting of CO_2_ into separate streams of CO and O_2_ using a ceria membrane reactor driven by concentrated radiation. The stable experimental results obtained under realistic high-flux conditions and the scalability of the simple tubular design demonstrate the viability of the solar membrane reactor technology for converting CO_2_ to fuels.

## Results and Discussion

**Figure 1:**
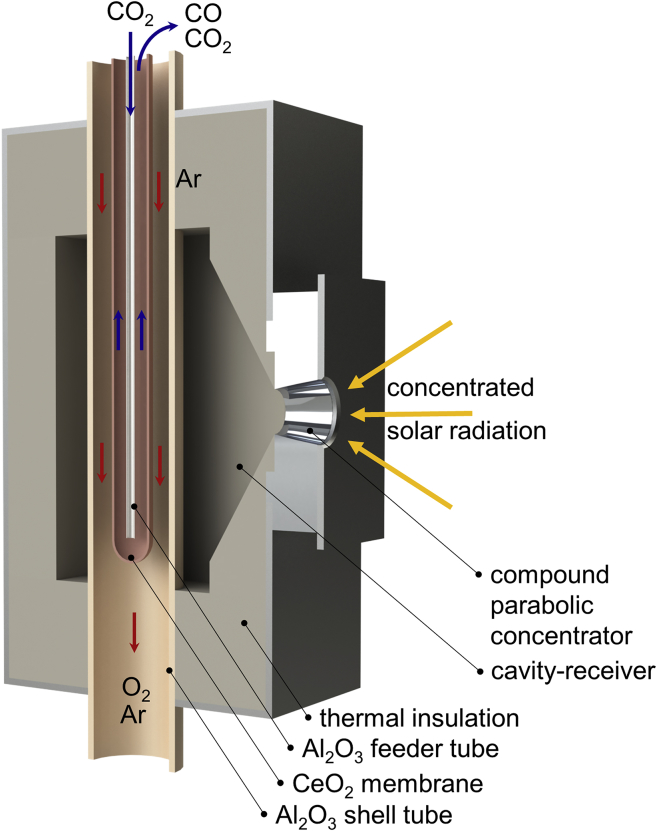
Scheme of the Solar Reactor Configuration The solar reactor comprises a cavity-receiver containing a capped tubular membrane, made of ceria, enclosed by a coaxial alumina tube. CO_2_ is supplied to the inner side (oxidation side) of the membrane, and a sweep inert gas is supplied to the outer side (reduction side) to control the oxygen partial pressure. Relative dimensions are not to scale.

The novel solar reactor configuration is shown in Figure 1. It consists of a thermally insulated cavity-receiver with a small aperture for the access of concentrated solar radiation. The cavity geometry enables efficient radiative capture by internal multiple reflections, approaching a blackbody absorber. The cavity contains a capped tubular membrane, made of ceria, enclosed by a coaxial alumina tube. CO_2_ is supplied to the inner side (oxidation side) of the membrane, and a sweep inert gas Ar is supplied to the outer side (reduction side) to control the oxygen partial pressure $p_{{\text{O}}_{2}}$. Both sides are operated at ambient total pressure. Though not demonstrated here, the analogous net splitting of H_2_O to produce H_2_ and O_2_ in separate streams can be achieved by supplying H_2_O instead of CO_2_.

**Figure 2:**
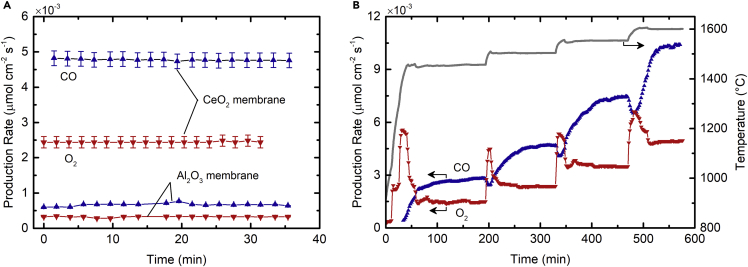
Transient and Steady-State Behavior of Specific Gas Production Rates (A) Steady-state specific production rates of CO and O_2_ during a CO_2_-splitting run at 1,500°C with a redox-active CeO_2_ membrane and a redox-inactive Al_2_O_3_ membrane. O_2_ is measured on the outer side of the CeO_2_ membrane and on the inner side of the Al_2_O_3_ membrane. Error bars are computed from device measurement uncertainties via error propagation (see Supplemental Information). (B) Specific production rates of CO and O_2_ with a CeO_2_ membrane over the course of stepwise increase in the temperature from 1,450°C to 1,600°C. Experimental conditions: 3,000–3,500 suns radiative flux, 25 mL min^−1^ CO_2_ (inner side), 200 mL min^−1^ Ar (outer side), 6 × 10^−5^ bar $p_{{\text{O}}_{2}}$, and ambient total pressure.

For all experimental runs reported here, the measured O_2_ and CO evolution rates confirmed a closed mass balance for 100% selectivity of CO_2_ to CO and O_2_ (molar ratio O_2_:CO = 0.51 ± 0.04, averaged for 21 runs), without any evidence of carbon deposition or any other byproducts. Figure 2A shows the specific production rates of CO and O_2_ over time at a nominal temperature of 1,500°C and $p_{{\text{O}}_{2}}$ of 6 × 10^−5^ bar on the outer side of the membrane, reached under steady-state operation for a radiative flux concentration of 3,000 suns (1 sun is equivalent to 1 kW/m^2^) and mass flow rates of 25 mL min^−1^ CO_2_ in the inner side and 200 mL min^−1^ Ar in the outer side (L denotes standard liters). Note that the reported $p_{{\text{O}}_{2}}$ refers to the Ar inlet flow, the minimum in the system. The mean CO production rate was 0.0048 μmol s^−1^ cm^−2^ and the molar conversion of CO_2_ was 0.82 mol%. For verification, the redox-active CeO_2_ membrane was replaced by a redox-inactive Al_2_O_3_ membrane, yielding both CO and O_2_ in the inner side due to CO_2_-thermolysis without O_2_ removal, and a CO production rate of only 0.0007 μmol s^−1^ cm^−2^—a factor of 7.1 lower. Thus, the CeO_2_ membrane shifted the thermodynamic equilibrium of CO_2_-thermolysis by thermochemically pumping oxygen along a chemical potential gradient across the membrane into an oxygen-lean sweep gas. Steady-state operation was observed for this 35 min run and for all other experiments performed, while the stable conditions suggested that the fuel production could be continued as confirmed for a similar 260 min steady-state run (Figure S1).

Figure 2B shows the CO and O_2_ production rates versus time over the temperature range from 1,450°C to 1,600°C. Temperatures are controlled by varying the radiative flux in the range of 3,000–3,500 suns. With each stepwise increase in temperature, a transient response followed by a steady-state product formation rate is observed due to the combined effect of thermodynamics and kinetics. Specifically, an increase in temperature shifts the equilibrium reduction extent of ceria (Equation 1) and drives the O_2_ release from the crystal lattice on both sides of the membrane. This leads to a temporal maximum in the O_2_ evolution rate at the outer side of the membrane. Concurrently, at the inner side of the membrane, the CO production rate initially drops because of recombination with the released O_2_ and then increases and levels off to twice the molar value of the O_2_ production rate as steady-state is reached in the exchange of oxygen ions, electrons (Ce^3+^/Ce^4+^ change), and vacancies. The mass balance is therefore closed. The differing timescales for the stabilization of CO and O_2_ are attributed to the different inlet flow rates of Ar and CO_2_ on either side of the membrane combined with the system time delay between the solar reactor exit and the gas measurement point downstream.

**Figure 3:**
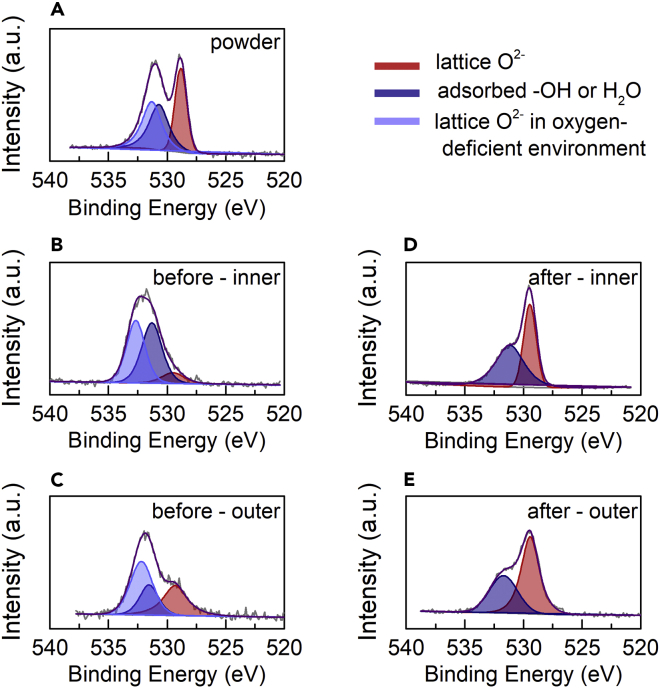
O 1s XPS Spectra between 540 and 525 eV (A–E) Pristine ceria powder (A), the ceria membrane inner surface analyzed before (B), the ceria membrane outer surface analyzed before (C), the ceria membrane inner surface analyzed after (D), and the ceria outer surface analyzed after (E) a CO_2_ splitting run of 443 min at 1,450°C–1,550°C and 6 × 10^−5^ bar O_2_. The XPS deconvolution peaks are attributed to the lattice oxygen (red), surface oxygen in the form of adsorbed −OH or H_2_O (dark blue), and lattice oxygen in oxygen-deficient regions (light blue), listed from lowest to highest binding energy. See also Figures S4 and S5.

Scanning electron microscopy images of the surface and cross-section of the ceria membrane before and after 443 min of reaction indicate that the morphological structure is preserved (Figure S2). Further, X-ray diffraction and energy dispersive spectroscopy confirm that the bulk composition of the ceria membranes is unchanged and matches that of the pristine ceria powder used to manufacture the membranes (Figure S3). Figure 3 shows the X-ray photoelectron spectroscopy (XPS) spectra for the O 1s orbital of ceria at the inner and outer surfaces of the membrane before and after the reaction, as well as for the pristine ceria powder used to manufacture the membranes. The XPS signal is deconvoluted into three peaks, attributed to lattice oxygen (O^2−^) at a binding energy of 529 eV, surface oxygen in the form of adsorbed −OH or H_2_O at a binding energy of ≈531 eV, and lattice oxygen in an oxygen-deficient environment with O^2−^ vacancies at a binding energy of ≈532 eV.^22^, ^23^, ^24^ The pristine ceria powder contains a mix of all three oxygen types. The membrane before reaction exhibits slightly higher O 1s binding energies compared to the ceria powder, indicative of O^2−^ vacancies formed during sintering, which varies spatially (Figures 3B and 3C) due to temperature gradients in the sintering process. After 443 min reaction at *T* = 1,450°C–1,550°C and $p_{{\text{O}}_{2}}$ = 6 × 10^−5^ bar, the binding energy is clearly shifted to lower values than those before reaction, indicative of the net consumption of O^2−^ vacancies due to the CO_2_ splitting reaction. Note that the difference in the peak intensity for the lattice oxygen is significantly higher than that caused by spatial variation in the O^2−^ vacancy concentration discussed above. The complete XPS analysis including the Ce 3d and C 1s spectra is provided in the Supplemental Information (Figures S4 and S5).

**Figure 4:**
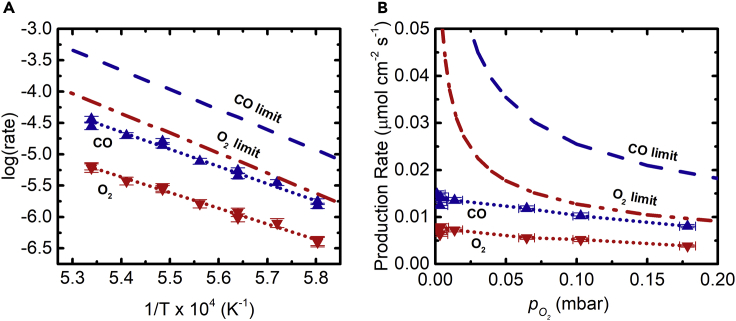
Thermodynamic Trends in Gas Production Rates (A and B) Average steady-state gas production rates as a function of: (A) temperature in the range 1,450°C–1,600°C with uncertainty of ±0.6°C at $p_{{\text{O}}_{2}}$ = 6 × 10^−5^; and (B) the partial pressure of O_2_ at the Ar inlet in the range 1 × 10^−6^–2 × 10^−4^ bar with uncertainty ±6 × 10^−6^ bar at *T* = 1,600°C. Dashed lines mark the thermodynamic limit. Dotted lines connect the data points for visual ease. See also Figure S7.

Figure 4 shows the measured steady-state gas production rates for CO and O_2_ as a function of: (a) temperature in the range 1,450°C–1,600°C at $p_{{\text{O}}_{2}}$ = 6 × 10^−5^ bar; and (b) $p_{{\text{O}}_{2}}$ in the range 1 × 10^−6^ to 2 × 10^−4^ bar at *T* = 1,600°C. Also indicated are the thermodynamic limits (derived in the Supplemental Information, Figure S6). The steady-state gas production rates are proportional to $\text{exp}\left(-E_{\text{A}}/RT\right)$, where the apparent activation energy *E*_A_ = 228 ± 9 kJ mol^−1^ CO matches the reaction enthalpy of 279 kJ mol^−1^ in this temperature range, suggesting conditions approaching thermodynamic equilibrium. The gas production rates decrease with $p_{{\text{O}}_{2}}$ as expected from the CO_2_-thermolysis equilibrium given by $K\left(T\right)=p_{\text{CO}}\cdot p_{{\text{O}}_{2}}^{1/2}/p_{{\text{CO}}_{2}}$ (Supplemental Information). However, particularly at $p_{{\text{O}}_{2}}$ < 5 × 10^−5^ bar, the thermodynamic limit is not approached. Interestingly, the specific CO and O_2_ production rates per unit area of membrane increase with the volumetric CO_2_ flow rate (Figure S7). This dependency presumably arises from mass transfer limitations, as the relatively low CO_2_ flow rate is unable to sweep away the CO product effectively on the inner side of the membrane. While the very high temperature and, in turn, relatively high activity of reactive oxygen vacancies at the inner surface of the membrane are expected to control the kinetics of the surface exchange reaction transferring oxygen from the gas phase into the solid phase, steady-state gas production rates may however be limited as well by this surface exchange. Thus, as anticipated,^4^ the overall kinetics were not controlled by solid-state diffusion within the crystal lattice of ceria, consistent with previous observations with a solar cavity-receiver containing a porous structure directly exposed to high-flux irradiation.^25^ This was indeed expected from the measured values of ambipolar diffusion coefficients of oxygen in ceria in the range 1.5 × 10^−5^–4 × 10^−4^ cm^2^ s^−1^ for 1,400°C–1,550°C,^4^ which translated to reduction times in the order of seconds for the length scales across the 0.5 mm-thick membrane. Thus, as far as solid-state diffusion is concerned, the transport of oxygen vacancies through the membrane is almost instantaneous compared with the timescales of Figure 2B.

One key metric for the solar reactor performance is the solar-to-fuel energy conversion efficiency, defined as the ratio of the heating value of the fuel produced to the solar energy input. The theoretical limit exceeds 40% at 1,600°C (see Thermodynamic Analysis in Supplemental Information), in agreement with a comparable thermodynamic study,^26^ but the measured value in this proof-of-concept prototype was less than 1% because no attempt was made to optimize the design. Straight-forward measures to boost the efficiency include the incorporation of an array of multiple tubular membranes inside the cavity-receiver for increasing the reaction surface and the recovery of the sensible heat of the outlet gas streams for preheating the inlet gas streams.

In sum, the feasibility of a solar membrane reactor for splitting CO_2_ into separate streams of CO and O_2_ has been demonstrated in terms of materials, selectivity, and stability. The design is simple, compact, modular, and scalable. The reaction rates were limited largely by mass and heat transfer, while the efficiency was limited mainly by the available membrane surface and the lack of heat recovery. Both limitations can be readily overcome through optimization and scale-up. Alternative membrane materials, e.g., doped ceria and perovskites, should exhibit rapid oxygen ion diffusivity at high operating temperatures while maintaining the thermochemical and structural stability of the membrane. Dual-membrane reactors for *in situ* removal of both O_2_ and CO produced by CO_2_-thermolysis should be explored for controlling the purity of the fuel produced. Notably, the large-scale implementation can be integrated to the concentrating solar tower and dish systems already established commercially. The solar production of CO from CO_2_ can be coupled to the synthesis of liquid hydrocarbon fuels for sustainable transportation.

## Experimental Procedures

### Materials

Cerium (IV) oxide (ceria, CeO_2_, powder, particle size < 5 μm, 99.9% purity), poly (oxy-1,4-phenylene sulfonyl-1,4-phenylene) (PES, (C_12_H_8_O_3_S)_n_, pellets), polyvinylpyrrolidone (PVP, (C_6_H_9_NO)_n_, powder, average M.W. 40,000), and 1-methyl-2-pyrrolidinone (NMP, C_5_H_9_NO, liquid, ≥ 99.0% purity) were from Sigma Aldrich. Coltogum high-temperature silicate adhesive (1,500°C maximum temperature) was from SFS unimarket AG. High-purity alumina adhesive (Resbond 989, Cotronics) was from Polytec PT GmbH. CO_2_ (99.998%), Ar (99.996%, 99.999%), He (99.999%), and calibration gas mixtures, i.e., 500 mol ppm O_2_ (99.999%) in Ar (99.999%), 500 mol ppm CO (99.997%) in Ar (99.999%), and 1,000 mol ppm CO (99.997%), 500 mol ppm CO_2_ (99.995%), 500 mol ppm N_2_ (99.999%), and 100 mol ppm O_2_ (99.999%) in Ar (99.999%) were from Messer Schweiz AG. According to the manufacturer, Ar (99.996%) contained <5 ppm O_2_ on volume basis, equivalent to a limiting $p_{{\text{O}}_{2}}$ < 5 × 10^−6^ bar.

### Membrane Fabrication

Capped tubular ceramic membranes were fabricated using a phase-inversion/sintering method.^27^, ^28^ PES (5.6–6.6 wt%) and PVP (0.4–0.5 wt%) were dissolved in NMP (22.7–26.6 wt%) by stirring magnetically at 30°C. Ceria powder (66.4–71.2 wt%) was gradually suspended in the polymer solution, and the slurry was stirred in a covered beaker for at least 24 hr. The slurry was coated onto a membrane template made of silicone tubing (High-Flexible tubing, 3 mm ID, 7 mm OD, RCT Reichelt Chemietechnik GMbH & Co.), and the assembly was placed in a water bath for phase inversion (unfiltered tap water coagulant). The membrane precursor was then dried in air at 20°C and sintered for 8 hr at 1,600°C (oven model HT 64/17, Nabertherm). The sintered membranes were 5–6 mm OD, 4–5 mm in inner diameter (ID), and 150–250 mm in length. The typical wall thickness was less than 0.5 mm. In addition to the redox-active ceria membranes produced in house, commercial redox-inactive Al_2_O_3_ membranes (Alsint 99.7, 7 mm OD, 5 mm ID, 250 mm length, Intertechno-Firag AG) were used.

### Solid-State Analysis

X-ray diffraction (XRD) was performed in the Bragg Brentano geometry using Cu Kα radiation (5-90° 2θ, 0.01° min^-1^ scan rate, 40 kV/40 mA output, Bruker D8 Advance). Basic structural data were obtained by multiphase Rietveld analysis (X’Pert HighScore). Scanning electron microscopy (SEM; 3 kV accelerating voltage, Hitachi, TM-1000) was used to analyze the membrane morphology. X-ray photoelectron spectroscopy (XPS) analyses were performed on a Phoibos 150 spectrometer calibrated at 84 eV against a clean Au(111) orientated single crystal. These analyses were carried out using a monochromatized Al Kα X-ray source with a photon energy of 1,486.7 eV and an energy resolution of about 0.8 meV. The sample area intercepted by X-rays was about 1.5 mm × 1.5 mm, and total pressures of the load lock and analysis chamber were 10^-8^ and 10^-11^ mbar, respectively. Survey (wide-scan) spectra and multiplex (narrow-scan) spectra of Ce 3d, O 1s, and C 1s were collected. The quantification and simulation of the experimental photo peaks were carried out using UNIFIT201. The line shapes used for curve fitting were a mix of Gaussian and Lorentian, and the background was fitted with the Shirley function.

### Solar Experimental Setup

The tubular ceria membrane was placed inside a larger coaxial Al_2_O_3_ tube (Alsint 99.7, OD 25 mm, ID 20 mm, MTC Haldenwanger) through which Ar sweep gas was fed in an annular flow. A smaller coaxial Al_2_O_3_ tube (Rubalit, OD 3 mm, ID 1.5 mm, CeramTec) was placed inside the tubular membrane near the closed end to feed CO_2_ in a countercurrent flow through the inner side of the membrane. This assembly was placed vertically inside a thermally insulated solar cavity-receiver with a 4 cm-diameter aperture. A compound parabolic concentrator of acceptance angle 45° was incorporated onto the aperture to boost the solar flux concentration by a factor of 2 and generate a more uniform directional distribution of concentrated radiation entering the cavity.^29^ Experimentation was performed at the High-Flux Solar Simulator (HFSS) of ETH Zurich: an array of seven Xe-arcs, close-coupled to truncated ellipsoidal reflectors, provided an external source of intense thermal radiation that closely approximated the heat transfer characteristics of highly concentrating solar systems. The radiative flux distribution at the focal plane was measured optically using a calibrated charge-coupled device camera focused on a Lambertian (diffusely reflecting) target. The solar radiative power input through the aperture was calculated by integration of the radiative flux over the aperture area and verified with a water calorimeter. Temperatures were measured at the outer surface of the reactor shell using B-type thermocouples. Ar and CO_2_ flow rates were regulated by electronic mass flow controllers (Bronkhorst F-201 C). Product gas composition was monitored on-line by gas chromatography (GC, Agilent 490 MicroGC).

### Experimental Runs

All volume flow rates are given at standard conditions (1 bar and 0°C). During a typical run, the reactor was heated by the HFSS with a radiative power input of 2.0–2.5 kW to the desired nominal temperature in the range 1,300°C–1,600°C. The CO_2_-splitting reaction was initiated by purging the membrane with 25 mL min^−1^ CO_2_ (inside, oxidation side) and 100–800 mL min^−1^ Ar (outside, reduction side). The value of $p_{{\text{O}}_{2}}$ in the Ar inlet flow included a small amount of air leakage into the system, as verified from the measured partial pressures of N_2_, and could be controlled by varying the Ar flow rate. The compositions of both gas streams exiting the solar reactor were analyzed simultaneously using two GCs. Steady-state was defined here as the condition at which the measured gas concentration was within 2% of the mean over the previous five consecutive measurements collected at a frequency of one every 2 min:

(Equation 3) $$\frac{\left|c_{i}\left(t_{n}\right)-\frac{1}{5}\sum_{j=n-5}^{n-1}c_{i}\left(t_{j}\right)\right|}{\frac{1}{5}\sum_{j=n-5}^{n-1}c_{i}\left(t_{j}\right)}\leq 0.02$$

where *c*_*i*_(*t*_*j*_) is the concentration of species *i* at time point *j*. Steady-state data were collected for at least 12 min, and the arithmetic mean was used to summarize the results at each experimental condition.

## Author Contributions

M.T., R.M., and A.S. designed the solar reactor. M.T. executed the experiments. R.M. and A.S. supervised the project.

## Supplemental Information

Document S1. Supplemental Text, Figures S1–S7, and Table S1

Document S2. Article plus Supplemental Information
