# Supplementary material for: Solar-Driven Thermochemical Splitting of CO2 and In Situ Separation of CO and O2 across a Ceria Redox Membrane Reactor
Source: Joule. 2017 Sep 6;1(1):146–54. doi: 10.1016/j.joule.2017.07.015 (PMC5632959; doi:10.1016/j.joule.2017.07.015)
Supplement: Document S1. Supplemental Text, Figures S1–S7, and Table S1 [file mmc1.pdf]

JOUL, Volume *1*

Supplemental Information

Solar-Driven Thermochemical Splitting of CO_2 and *In Situ* Separation of CO and O_2 across a Ceria Redox Membrane Reactor

Maria Tou, Ronald Michalsky, and Aldo Steinfeld

Supplemental Text

Reference experiments of direct thermolysis with a redox-inactive Al_2O_3 membrane were conducted at 1300, 1400, and 1500°C. No O_2 production was detected on the outer (reduction) side. In the inner (oxidation) side, the average O_2:CO molar ratio was 0.49 ± 0.03, indicating a closed mass balance. The measured CO_2 conversion matched the magnitude expected for equilibrium thermolysis (see section Thermodynamic Analysis). Small discrepancies arose presumably from non-uniform temperatures in the reaction zone as well as O_2/CO partial recombination upon cooling the product gas to ambient temperature. The longest CO_2-splitting experiment conducted in this study maintained steady-state for 260 minutes, as shown in Fig. S1. Once steady state was reached at 1475°C, the production rates of CO and O_2 remained constant, thus indicating stable reactor performance. The O_2:CO ratio was 0.52 ± 0.05.

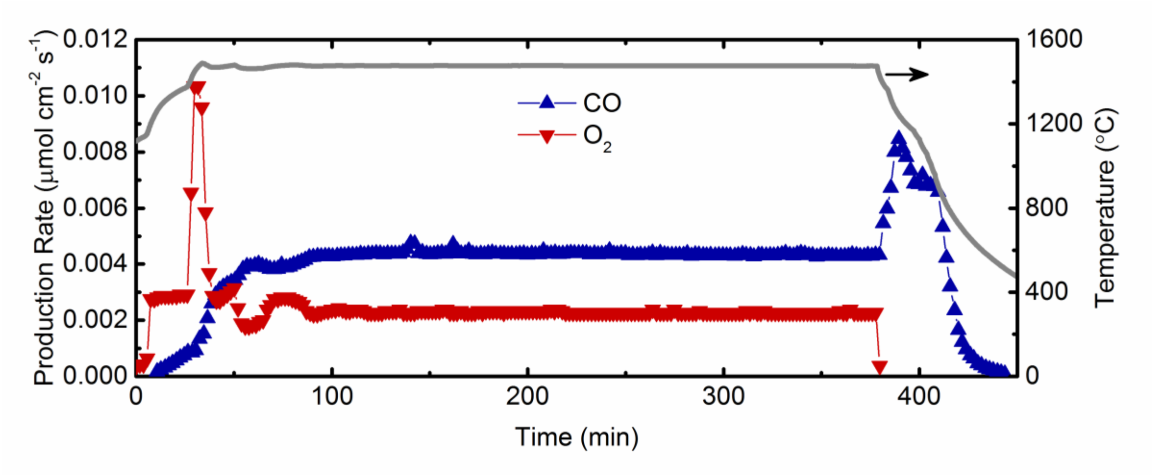

Fig. S1. Solar experimental run for CO_2 splitting with a ceria redox membrane.

<u>Materials characterization</u>

Figures S2a and S2b show SEMs of the membrane surface before and after a CO_2 splitting run, respectively. The dense surface exhibits some defects visible as dark spots on or between ceria grains, attributed to the pore structure of grains that have not fully sintered. Contamination was considered negligible as verified by energy dispersive spectroscopy (EDS). In general, pores do not pose a priori a problem provided they are unconnected and do not extend through the entire cross-section. This characteristic is necessary to keep the oxidizing and reducing streams separate and is seen in the membrane cross-section in Figs. S2c and S2d before and after a CO_2 splitting run, respectively. Mass transfer across the membrane is exclusively by diffusion of oxygen ions (O^{2-}) through the crystal lattice. The inner and outer surfaces of the tubular membrane can be distinguished in the SEM images by the curvature of the cross-section. The larger pores on the outer surface can be attributed to the phase-inversion synthesis technique and the inward shrinkage during sintering. Membrane thickness is typically less than 0.5 mm, which is below the critical length where O^{2-} diffusion in ceria becomes rate limiting.[4]

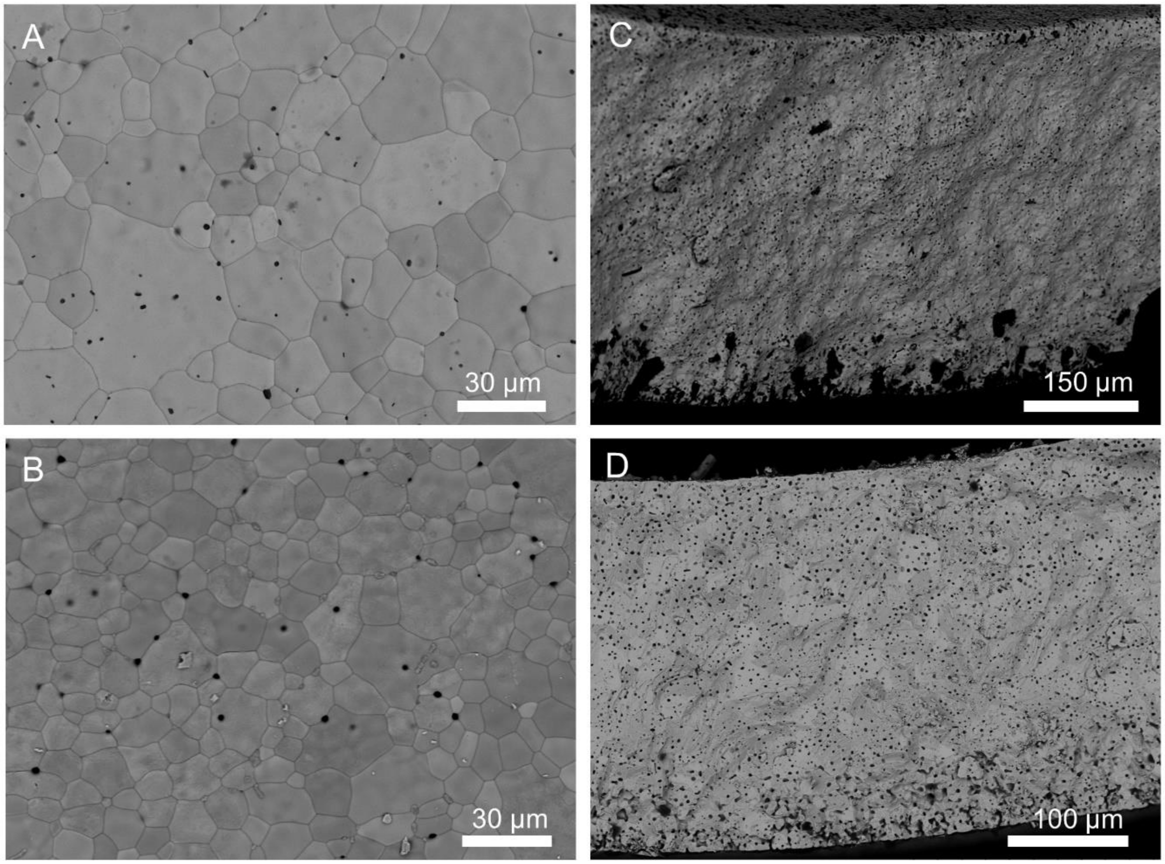

Fig. S2. SEM images of the ceria membrane surface (a, b) and cross section (c, d) analyzed before (a, c) and after (b, d) a CO_2 splitting run in the solar reactor.

Figure S3 shows the XRD spectra of pristine (as-purchased) ceria powder (i), and of the ceria membrane before (ii) and after (iii) a CO_2 splitting run in the solar reactor. The membrane composition is unchanged before and after reaction and matches the pristine ceria powder. Diffraction peaks are narrower in the spectra corresponding to membranes than in those from the powder due to the sintering of particles during fabrication.

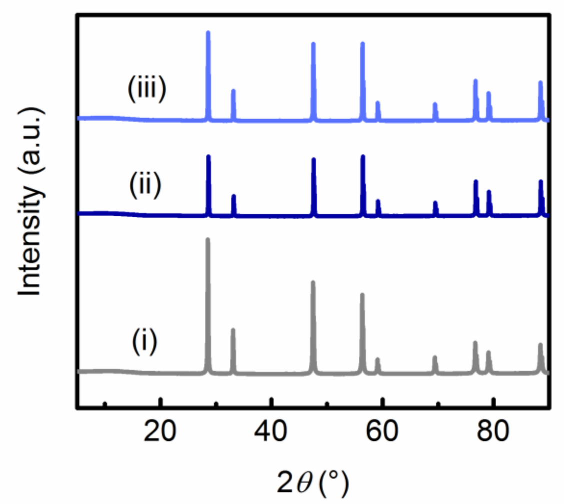

Fig. S3. XRD spectra of pristine ceria powder (i), and of the ceria membrane before (ii) and after (iii) a CO_2 splitting run in the solar reactor.

Figure S4 shows the XPS spectra for the C 1s orbital of ceria at the inner and outer surfaces of the membrane before and after reaction, as well as for the pristine ceria powder used to manufacture the membranes. The primary peak at ~285 eV is adventitious carbon attributed to adsorbed carbonaceous species from air.[30,31] Peaks appearing at ~286 eV and at ~288 eV are attributed to oxidized carbonaceous species also arising from exposure to air, where the higher binding energy peak is associated with carbonates.[30-32] Carbonaceous species were detected in all samples, which was expected as samples were stored in air at ambient conditions, without any cleaning before analysis.

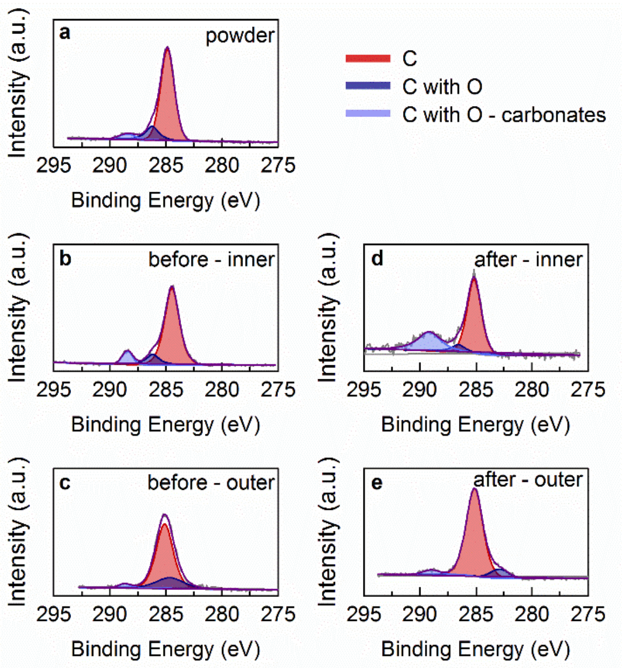

Fig. S4. C 1s XPS spectra between 295 and 275 eV of (a) $CeO_{2-\delta}$ powder as-purchased, and of the $CeO_{2-\delta}$ membrane (b, d) outer surface, and (c, e) inner surface, analyzed (b, c) before and (d, e) after CO_2 splitting for 443 min at 1450-1550°C and 6×10^{-5} bar O_2. The XPS deconvolution peaks are attributed to adventitious carbon (red) and oxidized carbon species (dark blue, light blue), where the higher binding energy peak is associated with carbonates (light blue).

Fig. S5 shows XPS spectra for the Ce 3d orbital of ceria at the inner and outer surfaces of the membrane before and after reaction, as well as for the pristine ceria powder used to manufacture the membranes. The XPS signal is deconvoluted into eight peaks, corresponding to four pairs of spin-orbit doublets, where peaks at ~897 and ~916 eV are attributed to Ce^{4+} and peaks at ~885 and ~903 eV are attributed to Ce^{3+}.[33-35] In general, the samples exhibited a mix of Ce^{4+} and Ce^{3+}, with Ce^{3+} as the relatively smaller fraction. The overall Ce 3d signal was small compared to O 1s across all samples.

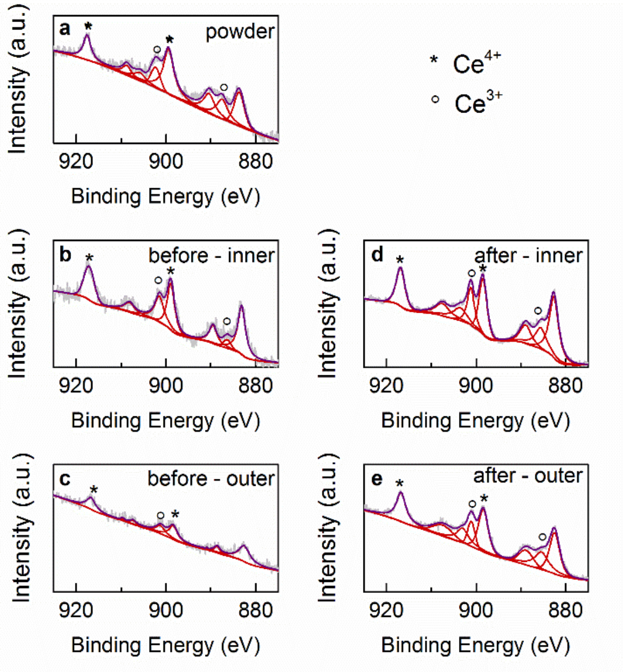

Fig. S5. Ce 3d XPS spectra between 925 and 875 eV of (a) $CeO_{2-\delta}$ powder as-purchased, and of the $CeO_{2-\delta}$ membrane (b, d) outer surface, and (c, e) inner surface, analyzed (b, c) before and (d, e) after CO_2 splitting for 443 min at 1450-1550°C and 6×10^{-5} bar O_2. Peaks attributed to Ce^{4+} and Ce^{3+} are denoted by asterisks (*) and open circles (°), respectively.

Thermodynamic Analysis

Assuming ideal gases for a reference state at 1 bar, the equilibrium constant of CO_2 thermolysis ($CO_2 = CO + ½O_2$) is given by

$$K(T) = \frac{p_{\mathrm{CO}}\sqrt{p_{\mathrm{O_2}}}}{p_{\mathrm{CO_2}}} = \exp\left(\frac{-\Delta G^{\circ}_{\mathrm{rxn}}}{RT}\right) \qquad \text{(Eq. S1)}$$

where p_i is the partial pressure of gas species i, $\Delta G^{\circ}_{\mathrm{rxn}}$ is the standard Gibbs free energy change, and R is the universal gas constant. $K(T)$ was computed using thermodynamic data from NIST JANAF tables (http://kinetics.nist.gov/janaf/). Assuming a feed of pure CO_2, the gas composition must further satisfy $p_{\mathrm{total}} = p_{\mathrm{CO_2,i}} = p_{\mathrm{CO_2}} + p_{\mathrm{CO}} + p_{\mathrm{O_2}}$. The fundamental concept of the semi-permeable redox membrane is that it can shift the CO_2 thermolysis equilibrium by removing O_2 from the inner (oxidation) side and transporting it to the outer (reduction) side. Thus, the presence of the membrane introduces an additional degree of freedom besides T and p_{total}, namely setting $p_{\mathrm{O_2}}$ at the inlet on the outer (reduction) side of the membrane. This can be accomplished by vacuum pumping or, as in this case, by a purging flow of Ar. In the counter-current arrangement, $p_{\mathrm{O_2}}$ exiting the inner side equilibrates with $p_{\mathrm{O_2}}$ entering the outer side of the membrane and the product composition can be determined from Eq. S1. Thus, the driving force of CO_2 splitting can be expressed as the difference in $p_{\mathrm{O_2}}$ between the CO_2 inlet and outlet, the former dependent

on temperature only and the latter dependent on the p_{O_2} set in the reduction inlet. This Δp_{O_2} driving force is shown in Fig. S6a as a function of T and p_{O_2} at the reduction inlet in the ranges T = 1200-1700°C and p_{O_2} = 10^{-7}-10^{-3} bar at p_{total} = 1 bar. Evidently, Δp_{O_2} increases with temperature because of its correlation with the thermolysis equilibrium constant. Negative values of Δp_{O_2} indicate unfavorable conditions where the membrane provides no benefit. The conversion of CO_2 at equilibrium is shown in Fig. S6b at the same T and p_{O_2} conditions. As expected, the conversion increases monotonically with temperature and decreases with p_{O_2}. Unlike in a two-step redox cycle, a lower oxidation temperature detrimentally affects the thermolytic driving force.

The solar-to-fuel energy conversion efficiency, $\eta_{\text{solar-to-fuel}}$, is defined for steady-state CO_2 splitting as:

$$\eta_{\text{solar-to-fuel}} = \frac{\dot{n}_{\text{CO}} \cdot \text{HV}_{\text{CO}}}{\dot{Q}_{\text{solar}}} \quad \text{(Eq. S2)}$$

where $\dot{n}_{CO}$ is the molar production rate of CO, HV_{CO} is the heating value of CO (HV_{CO} = 283 kJ/mol) and $\dot{Q}_{solar}$ is the total solar radiative power input. $\dot{n}_{CO}$ is calculated from the equilibrium conversion of CO_2 at T, p_{O_2}, and p_{total} conditions (Fig. S6b). $\dot{Q}_{solar}$ accounts for the reaction enthalpy, sensible heating of feed gases, re-radiation losses through the aperture, heat losses through the walls, as well as the power required for the separation of product gases:

$$\dot{Q}_{\text{solar}} = \dot{Q}_{\text{rxn}} + \dot{Q}_{\text{sensible}} + \dot{Q}_{\text{reradiation}} + \dot{Q}_{\text{wall}} + \dot{Q}_{\text{separation}} \quad \text{(Eq. S3)}$$

The heat required for the reaction enthalpy is:

$$\dot{Q}_{\text{rxn}} = \dot{n}_{\text{CO}} \Delta H_{\text{rxn}}(T) \quad \text{(Eq. S4)}$$

The sensible heat term includes the power required to heat both the CO_2 and Ar gas streams from room temperature T_0 to the operating temperature T, less the heat recovered from the exiting hot gases with a heat exchanger efficacy η_{HEX}:[26,36-38]

$$\dot{Q}_{\text{sensible}} = \left[\dot{n}_{\text{CO}_2,i}\left(h_{\text{CO}_2}(T) - h_{\text{CO}_2}(T_0)\right) + \dot{n}_{\text{Ar},i}\left(h_{\text{Ar}}(T) - h_{\text{Ar}}(T_0)\right)\right](1-\eta_{\text{HEX}}) \quad \text{(Eq. S5)}$$

The change in the flow thermal properties due to the change in composition is neglected. The solar cavity-receiver is assumed to be a perfectly insulated blackbody absorber. Thus,[39]

$$\dot{Q}_{\text{reradiation}} = \left(\frac{\sigma T^4}{IC}\right)\dot{Q}_{\text{solar}} \quad \text{(Eq. S6)}$$

where I is the direct normal solar irradiation (DNI), C is the solar concentration ratio, and σ is the Stefan-Boltzmann constant. Heat loss through walls is proportional to the solar radiative power absorbed by the cavity, represented by a loss factor F,

$$\dot{Q}_{\text{wall}} = F\left(\dot{Q}_{\text{solar}} - \dot{Q}_{\text{reradiation}}\right) \quad \text{(Eq. S7)}$$

The energy required for product gas separation was computed from the minimum work based on the Gibbs free energy of mixing ideal gases in a binary mixture:

$$\dot{Q}_{\text{separation}} = \frac{RT}{\eta_{\text{Carnot}}}\left[\left(\dot{n}_{1,\text{f}} \ln x_{1,\text{f}} + \dot{n}_{2,\text{f}} \ln x_{2,\text{f}}\right) - \left(\dot{n}_{1,\text{i}} \ln x_{1,\text{i}} + \dot{n}_{2,\text{i}} \ln x_{2,\text{i}}\right)\right] \quad \text{(Eq. S8)}$$

where $x_{n,i/f}$ is the molar fraction of the nth component at the initial or final mixture composition. Carnot efficiency is assumed for the conversion of heat to work. The parametric values used in this analysis are summarized in Table S1. Fig. S6c shows $\eta_{\text{solar-to-fuel}}$ as a function of temperature in the range 1200-1700°C for p_{O_2} = 10^{-7}-10^{-3} bar and p_{total} = 1 bar. Evidently, $\eta_{\text{solar-to-fuel}}$ correlates with the CO_2 conversion. However, contrary to the trend in the CO_2 conversion, the incremental increase of $\eta_{\text{solar-to-fuel}}$ decreases with

increasing T and decreasing p_{O_2} levels. The results are in good agreement with previous studies making comparable assumptions.[26,36,37,40]

Table S1. Baseline parameters for the calculation of $\eta_{\text{solar-to-fuel}}$

Direct normal solar irradiation, I	1 kW m^{-2}
Solar concentration ratio, C	3000
Temperature, T	1500°C
Partial pressure of O_2 in Ar feed, p_{O_2}	10^{-5} bar
Total pressure, p_{total}	1 bar
Heat recovery efficacy, η_{HEX}	0.95
Ambient temperature, T_0	25°C
Heat loss factor, F	0.2
CO purity after separation	0.38

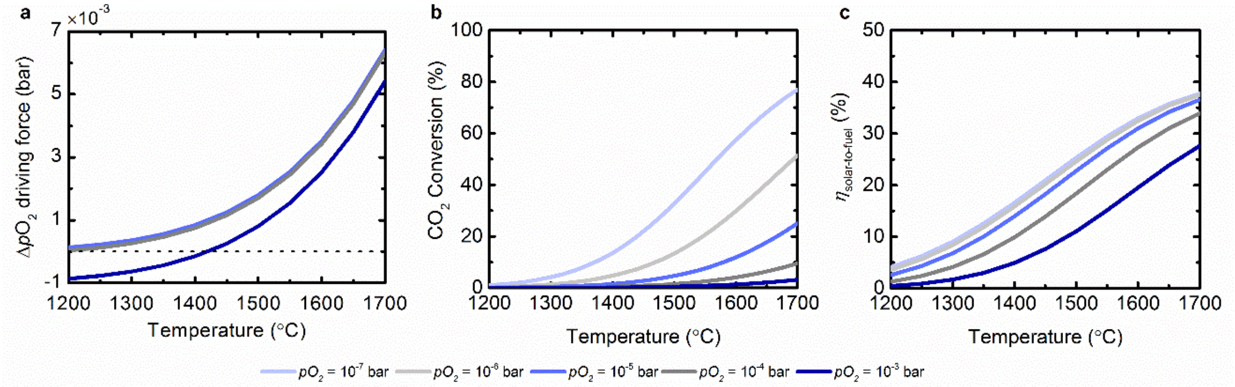

Fig. S6. For the conditions in the ranges T = 1200-1700°C and $p_{O_2} = 10^{-7}$-10^{-3} bar at p_{total} = 1 bar: (a) Δp_{O_2} driving force for CO_2 splitting; (b) CO_2 conversion at equilibrium; and (c) the solar-to-fuel energy conversion efficiency. The parameter is p_{O_2} at the Ar inlet on the reduction side of the redox membrane.

A sensitivity analysis on $\eta_{\text{solar-to-fuel}}$ was conducted to identify critical parameters by varying their value by ±20%. η_{HEX} had the largest effect: a 20% reduction resulted in $\Delta\eta_{\text{solar-to-fuel}}$ = -15% while perfect heat recovery led to $\Delta\eta_{\text{solar-to-fuel}}$ = +40% relative to the base case. Varying the other parameters changed $\eta_{\text{solar-to-fuel}}$ by less than 2%. To separate O_2 and recycle Ar, waste heat can be employed to drive a thermochemical redox cycle with perovskites.[41] Depending on the downstream use of CO, there may not be a need to separate it from unreacted CO_2, especially for high CO_2 conversions, as shown for syngas-to-liquid processing for the synthesis of kerosene[42] and methanol.[43]

Steady-state gas production rate limitations

Experimental CO_2 conversion rates deviated from the equilibrium values, especially at low p_{O_2}. The thermodynamic analysis assumes plug flow, setting an ideal upper limit for the gas production rates.

However, because of the boundary layer formation, the p_{O_2} near the walls may be greater than in the bulk, which could account in part for the experimental disparity from the calculated limit. We speculate that a possible cause for greater deviations at low p_{O_2} was mass transfer limitation on the inner (oxidation) side, because the constant CO_2 flow rate could not adequately remove CO from the reaction site. To check this hypothesis, an additional set of experiments were conducted at 1600°C with a fixed Ar flow rate and varied CO_2 flowrates. Fig. S7 shows the average steady-state gas production rates vs. p_{O_2} for various CO_2 flowrates. Dashed lines mark the thermodynamic limit; dotted lines connect the data points for visual ease. For clarity, the abscissa is plotted on a logarithm scale. This figure is a reproduction of the data of Fig. 4b, obtained for 25 mL min^{-1} CO_2, overlaid with hollow symbols representing the additional data points at 50, 100, and 200 mL min^{-1} CO_2. Higher CO_2 flowrates resulted in an increase in the production towards the thermodynamic limit, indicative of mass transfer limitation. However, similar results would be expected of a surface exchange reaction limited conversion. The maximum CO production rate across all experiments was 0.024 µmol s^{-1} per cm^2 of membrane, measured at T = 1600°C and p_{O_2} = 3×10^{-6} bar.

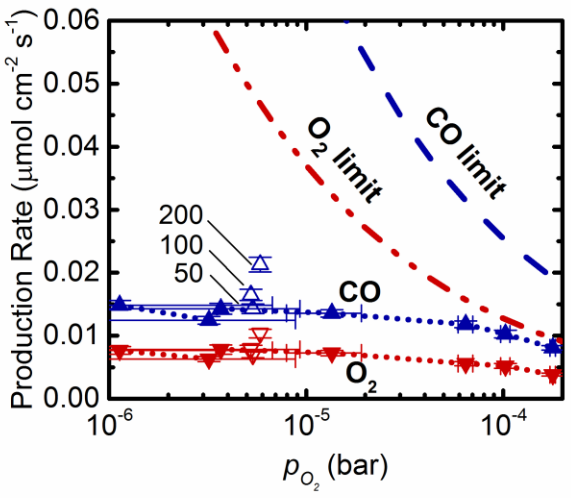

Fig. S7. Average steady-state gas production rates at 1600°C for various CO_2 flow rates versus the partial pressure of O_2 at the Ar inlet (in the range 1×10^{-6} - 2×10^{-4} bar with uncertainty ±6×10^{-6} bar). Error bars are computed from device measurement uncertainties via error propagation. Solid symbols represent data at 25 mL min^{-1} CO_2, originally shown in Fig. 4b. Dotted lines connect these data points for visual ease. Hollow symbols represent data points at 50, 100, and 200 mL min^{-1} CO_2, in order of increasing production rate. Dashed lines mark the thermodynamic limit.

Error analysis

The membrane was tested for gas-tightness by purging with CO_2 (inner side) and Ar (outer side) at ambient temperature. The CO_2 concentration in the outer side was below 10 ppm – the GC's detection limit. An O_2 leak rate of up to 0.015 µmol O_2 s^{-1} summed over both sides was derived from ambient air, which was taken into account in the measurement of the O_2 production rate. To distinguish the O_2 derived from CO_2, the small amount of ambient O_2 entering the system was calculated from the measured partial pressure of N_2. No CO was ever measured on the outer (reduction) side, even at high temperatures. The specific molar production rates of CO and O_2 were calculated as:

$$\dot{n}_{\mathrm{CO}} = \frac{\rho_{\mathrm{CO}} \dot{v}_{\mathrm{CO_2}}}{A_{\mathrm{membrane}} M_{\mathrm{CO}}} x_{\mathrm{CO}} \qquad \text{(Eq. S9)}$$

$$\dot{n}_{O_2} = \frac{\rho_{O_2} \dot{v}_{Ar}}{A_{membrane} M_{O_2}} x_{O_2} \qquad \text{(Eq. S10)}$$

where $A_{membrane}$ is the active area of the membrane, x_i is the volume fraction of species i on the appropriate side measured by the GC, $\dot{v}_i$ is the standard volumetric flow rate of gas i, and ρ_i and M_i are the standard density and molar mass of species i. Error bars for $\dot{n}_{CO}$ and $\dot{n}_{O2}$ were calculated using error propagation analysis. The uncertainties were quantified for each measured value, namely: x_i, v_i, $A_{membrane}$. For the GC measurements, uncertainty was taken to be the precision limit of 10 ppm. The uncertainty in the mass flow controllers was reported by Bronkhorst as: $\delta\dot{v} = (0.5\%\mathrm{Rd}) + (0.1\%\mathrm{FS})$, where the first term refers to the percentage of the reading (Rd) and the second refers to the percentage of the full-scale (FS) capacity of the mass flow controller (Bronkhorst Datasheet F-201CV). The same error propagation approach was used to evaluate the uncertainty in the molar ratio O_2:CO,

$$\delta\left(\frac{\dot{n}_{O_2}}{\dot{n}_{CO}}\right) = \frac{\dot{n}_{O_2}}{\dot{n}_{CO}}\left[\left(\frac{\delta\dot{n}_{O_2}}{\dot{n}_{O_2}}\right)^2 + \left(\frac{\delta\dot{n}_{CO}}{\dot{n}_{CO}}\right)^2\right]^{1/2} \qquad \text{(Eq. S11)}$$

Supplemental References

30 Barr, T.L., and Seal, S. (1995). Nature of the use of adventitious carbon as a binding energy standard. Journal of Vacuum Science & Technology A: Vacuum, Surfaces, and Films *13*, 1239-1246.

31 Batis, N.H., Delichere, P., and Batis, H. (2005). Physicochemical and catalytic properties in methane combustion of La1−xCaxMnO3±y (0≤x≤1; −0.04≤y≤0.24) perovskite-type oxide. Applied Catalysis A: General *282*, 173-180.

32 Moulder, J.F. (1992). Handbook of X-ray Photoelectron Spectroscopy: A Reference Book of Standard Spectra for Identification and Interpretation of XPS Data (Physical Electronics Division, Perkin-Elmer Corporation).

33 Xiao, W., Guo, Q., and Wang, E.G. (2003). Transformation of CeO2(1 1 1) to Ce2O3(0 0 0 1) films. Chemical Physics Letters *368*, 527-531.

34 Younis, A., Chu, D., and Li, S. (2012). Oxygen level: the dominant of resistive switching characteristics in cerium oxide thin films. Journal of Physics D: Applied Physics *45*, 355101.

35 Zhang, J., Zhao, H., Wei, F., Yang, M., Yang, Z., Chen, Q., and Chen, J. (2014). Resistive switching behaviour of highly epitaxial CeO2thin film for memory application. physica status solidi (RRL) - Rapid Research Letters *8*, 95-99.

36 Bader, R., Venstrom, L.J., Davidson, J.H., and Lipiński, W. (2013). Thermodynamic Analysis of Isothermal Redox Cycling of Ceria for Solar Fuel Production. Energy & Fuels *27*, 5533-5544.

37 Ermanoski, I., Miller, J.E., and Allendorf, M.D. (2014). Efficiency maximization in solar-thermochemical fuel production: challenging the concept of isothermal water splitting. Phys Chem Chem Phys *16*, 8418-8427.

38 Venstrom, L.J., De Smith, R.M., Hao, Y., Haile, S.M., and Davidson, J.H. (2014). Efficient Splitting of CO2in an Isothermal Redox Cycle Based on Ceria. Energy & Fuels *28*, 2732-2742.

39 Steinfeld, A., and Palumbo, R. (2001). Solar Thermochemical Process Technology. In Encyclopedia of Physical Science and Technology (Academic Press), pp. 237-256.

40 Wang, H., Hao, Y., and Kong, H. (2015). Thermodynamic study on solar thermochemical fuel production with oxygen permeation membrane reactors. International Journal of Energy Research *39*, 1790-1799.

41 Ezbiri, M., Allen, K.M., Galvez, M.E., Michalsky, R., and Steinfeld, A. (2015). Design Principles of Perovskites for Thermochemical Oxygen Separation. ChemSusChem *8*, 1966-1971.

42 Marxer, D., Furler, P., Scheffe, J., Geerlings, H., Falter, C., Batteiger, V., Sizmann, A., and Steinfeld, A. (2015). Demonstration of the Entire Production Chain to Renewable Kerosene via Solar Thermochemical Splitting of H2O and CO2. Energy & Fuels *29*, 3241-3250.

43 Ash-Kurlander, U., Martin, O., Fontana, L.D., Patil, V.R., Bernegger, M., Mondelli, C., Pérez-Ramírez, J., and Steinfeld, A. (2016). Impact of Daily Startup-Shutdown Conditions on the Production of Solar Methanol over a Commercial Cu-ZnO-Al2O3Catalyst. Energy Technology *4*, 565-572.
