# Supplementary material for: Solar-Driven Thermochemical Splitting of CO2 and In Situ Separation of CO and O2 across a Ceria Redox Membrane Reactor
Source: Joule. 2017 Sep 6;1(1):146–54. doi: 10.1016/j.joule.2017.07.015 (PMC5632959; doi:10.1016/j.joule.2017.07.015)
Supplement: Document S2. Article plus Supplemental Information [file mmc2.pdf]

Joule

CellPress

Article

Solar-Driven Thermochemical Splitting of CO_2 and *In Situ* Separation of CO and O_2 across a Ceria Redox Membrane Reactor

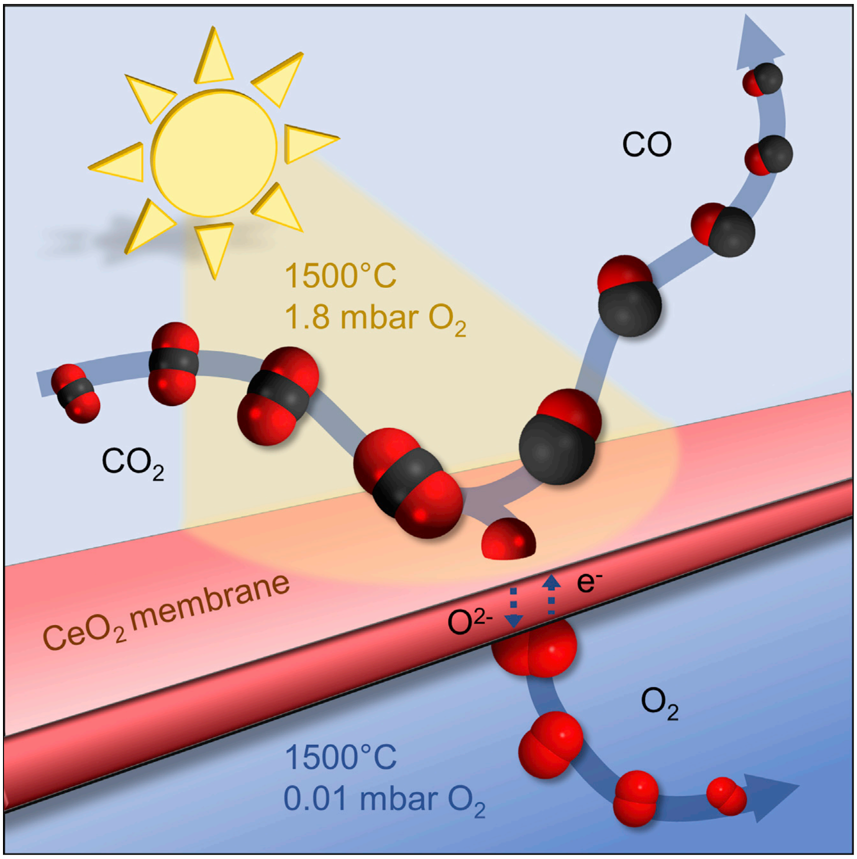

Conversion of CO_2 into fuels via a solar-driven thermochemical process enables the efficient production of sustainable transportation fuels. The solar reactor continuously splits CO_2 across a redox membrane into separate streams of CO and O_2 using concentrated solar radiation. The stable experimental results and the theoretical solar-to-fuel energy conversion efficiency support the viability of the modular solar reactor technology for converting CO_2 to fuels at a large scale by integration with the established concentrating solar tower and dish systems.

Maria Tou, Ronald Michalsky, Aldo Steinfeld

michalskyr@ethz.ch (R.M.)
aldo.steinfeld@ethz.ch (A.S.)

HIGHLIGHTS

Concentrated solar energy drives the thermochemical splitting of CO_2 to CO and O_2

Single-step continuous process is performed across a redox ceria membrane

Solar reactor is operated under steady-state isothermal/isobaric stable conditions

Total selectivity and *in situ* separation is obtained for products CO and O_2

Tou et al., Joule *1*, 146–154
September 6, 2017 © 2017 The Author(s).
Published by Elsevier Inc.
http://dx.doi.org/10.1016/j.joule.2017.07.015

CrossMark

Joule

CellPress

Article

Solar-Driven Thermochemical Splitting of CO_2 and *In Situ* Separation of CO and O_2 across a Ceria Redox Membrane Reactor

Maria Tou,[1] Ronald Michalsky,[1,*] and Aldo Steinfeld[1,2,*]

SUMMARY

Splitting CO_2 with a thermochemical redox cycle utilizes the entire solar spectrum and provides a favorable path to the synthesis of solar fuels at high rates and efficiencies. However, the temperature/pressure swing commonly applied between reduction and oxidation steps incurs irreversible energy losses and severe material stresses. Here, we experimentally demonstrate for the first time the single-step continuous splitting of CO_2 into separate streams of CO and O_2 under steady-state isothermal/isobaric conditions. This is accomplished using a solar-driven ceria membrane reactor conducting oxygen ions, electrons, and vacancies induced by the oxygen chemical potential gradient. Guided by the limitations imposed by thermodynamic equilibrium of CO_2 thermolysis, we operated the solar reactor at 1,600°C, $3 \cdot 10^{-6}$ bar p_{O_2} and 3,500 suns radiation, yielding total selectivity of CO_2 to CO + $^1/_2O_2$ with a conversion rate of 0.024 μmol$\cdot$s^{-1} per cm^2 membrane. The dynamics of the oxygen vacancy exchange, tracked by GC and XPS, further validated stable fuel production.

Context & Scale

Solar technologies that facilitate the efficient conversion of CO_2 into fuels offer a sustainable path to the production of renewable drop-in hydrocarbons for the transportation sector, especially for aviation, without the need to replace the existing massive global infrastructure for their storage, distribution, and consumption. We pursue the direct use of concentrated solar radiation as the source of high-temperature process heat to thermochemically split CO_2 into separate streams of CO and O_2. This is accomplished via a single-step continuous process across a redox membrane using a compact solar reactor design. The stable steady-state experimental results obtained with total selectivity under realistic high-flux operational conditions, along with the potential for high solar-to-fuel energy conversion efficiencies, support the viability of the modular membrane reactor technology for converting CO_2 to fuels at a large scale by integration with the established concentrating solar tower and dish systems.

INTRODUCTION

Developing solar technologies for converting CO_2 into fuels has become a grand energy challenge, as it closes the anthropogenic carbon cycle and leads to the production of sustainable transportation fuels.[1,2] The thermochemical approach is particularly appealing because the entire solar spectrum is utilized in the form of high-temperature process heat to drive a cycle based on metal oxide redox reactions.[3] Nonstoichiometric ceria ($CeO_{2-\delta}$) has emerged as an attractive redox active material because of its crystallographic stability and fast oxygen ion diffusivity.[4–6] The two-step redox cycle is represented by:

$$CeO_{2-\delta_{ox}} \rightarrow CeO_{2-\delta_{red}} + \frac{\Delta\delta}{2} O_2 \qquad \text{(Equation 1)}$$

$$CeO_{2-\delta_{red}} + \Delta\delta\, CO_2 \rightarrow CeO_{2-\delta_{ox}} + \Delta\delta\, CO \qquad \text{(Equation 2)}$$

In the endothermic reduction step driven by concentrated solar energy, Equation 1, $CeO_{2-\delta_{ox}}$ is reduced to $CeO_{2-\delta_{red}}$ and O_2 is liberated. The total number of oxygen vacancies formed in the ceria crystal lattice sets the upper limit for the specific oxygen exchange capacity, given by $\Delta\delta = \delta_{red} - \delta_{ox}$. In the exothermic oxidation step, Equation 2, $CeO_{2-\delta_{red}}$ is re-oxidized by CO_2 to generate CO. In principle, the redox cycle can be operated under a temperature/pressure-swing mode to control $\Delta\delta$ and thereby the fuel yield per cycle,[7] but these swings induce significant material stresses and energy irreversibilities.[8–11] Both temperature and pressure swings can be completely eliminated by means of a ceria membrane reactor that establishes a spatial separation between the reduction and oxidation sites and conducts oxygen ions, electrons, and vacancies driven by the oxygen chemical potential gradient across the membrane. With such an

 © 2017 The Author(s). Published by Elsevier Inc.
This is an open access article under the CC BY license (http://creativecommons.org/licenses/by/4.0/).

arrangement, both steps of the redox cycle can be performed simultaneously and continuously at isothermal/isobaric steady-state conditions. This in turn enables the design of a compacter solar reactor. Pioneering studies on solar thermal membrane reactors for splitting H_2O were conducted by Fletcher and co-workers.[12,13] Previous experimental studies with membrane reactors were non-solar and made use of a sacrificial reducing agent, such as CH_4,[14–17] CO,[18,19] or H_2[20,21] to scavenge the oxygen produced, thus failing to accomplish the net splitting of CO_2, i.e., $CO_2 \rightarrow CO + {}^1/_2\, O_2$. Here, we experimentally demonstrate for the first time the splitting of CO_2 into separate streams of CO and O_2 using a ceria membrane reactor driven by concentrated radiation. The stable experimental results obtained under realistic high-flux conditions and the scalability of the simple tubular design demonstrate the viability of the solar membrane reactor technology for converting CO_2 to fuels.

RESULTS AND DISCUSSION

The novel solar reactor configuration is shown in Figure 1. It consists of a thermally insulated cavity-receiver with a small aperture for the access of concentrated solar radiation. The cavity geometry enables efficient radiative capture by internal multiple reflections, approaching a blackbody absorber. The cavity contains a capped tubular membrane, made of ceria, enclosed by a coaxial alumina tube. CO_2 is supplied to the inner side (oxidation side) of the membrane, and a sweep inert gas Ar is supplied to the outer side (reduction side) to control the oxygen partial pressure p_{O_2}. Both sides are operated at ambient total pressure. Though not demonstrated here, the analogous net splitting of H_2O to produce H_2 and O_2 in separate streams can be achieved by supplying H_2O instead of CO_2.

For all experimental runs reported here, the measured O_2 and CO evolution rates confirmed a closed mass balance for 100% selectivity of CO_2 to CO and O_2 (molar ratio O_2:CO = 0.51 $\pm$ 0.04, averaged for 21 runs), without any evidence of carbon deposition or any other byproducts. Figure 2A shows the specific production rates of CO and O_2 over time at a nominal temperature of 1,500°C and p_{O_2} of 6×10^{-5} bar on the outer side of the membrane, reached under steady-state operation for a radiative flux concentration of 3,000 suns (1 sun is equivalent to 1 kW/m^2) and mass flow rates of 25 mL min^{-1} CO_2 in the inner side and 200 mL min^{-1} Ar in the outer side (L denotes standard liters). Note that the reported p_{O_2} refers to the Ar inlet flow, the minimum in the system. The mean CO production rate was 0.0048 μmol s^{-1} cm^{-2} and the molar conversion of CO_2 was 0.82 mol%. For verification, the redox-active CeO_2 membrane was replaced by a redox-inactive Al_2O_3 membrane, yielding both CO and O_2 in the inner side due to CO_2-thermolysis without O_2 removal, and a CO production rate of only 0.0007 μmol s^{-1} cm^{-2}—a factor of 7.1 lower. Thus, the CeO_2 membrane shifted the thermodynamic equilibrium of CO_2-thermolysis by thermochemically pumping oxygen along a chemical potential gradient across the membrane into an oxygen-lean sweep gas. Steady-state operation was observed for this 35 min run and for all other experiments performed, while the stable conditions suggested that the fuel production could be continued as confirmed for a similar 260 min steady-state run (Figure S1).

Figure 2B shows the CO and O_2 production rates versus time over the temperature range from 1,450°C to 1,600°C. Temperatures are controlled by varying the radiative flux in the range of 3,000–3,500 suns. With each stepwise increase in temperature, a transient response followed by a steady-state product formation rate is observed due to the combined effect of thermodynamics and kinetics. Specifically, an increase in temperature shifts the equilibrium reduction extent of ceria

[1]Department of Mechanical and Process Engineering, ETH Zürich, 8092 Zürich, Switzerland

[2]Lead Contact

*Correspondence: michalskyr@ethz.ch (R.M.), aldo.steinfeld@ethz.ch (A.S.)

http://dx.doi.org/10.1016/j.joule.2017.07.015

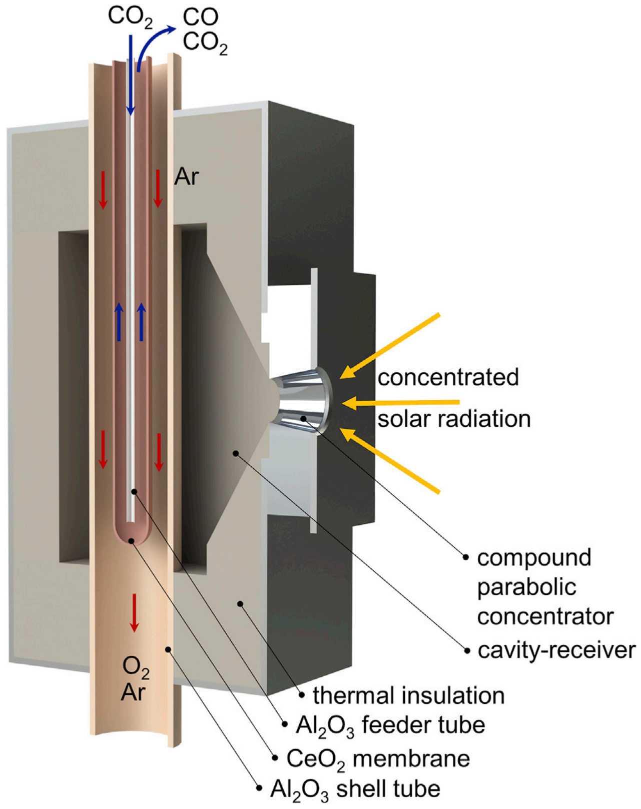

Figure 1. Scheme of the Solar Reactor Configuration

The solar reactor comprises a cavity-receiver containing a capped tubular membrane, made of ceria, enclosed by a coaxial alumina tube. CO_2 is supplied to the inner side (oxidation side) of the membrane, and a sweep inert gas is supplied to the outer side (reduction side) to control the oxygen partial pressure. Relative dimensions are not to scale.

(Equation 1) and drives the O_2 release from the crystal lattice on both sides of the membrane. This leads to a temporal maximum in the O_2 evolution rate at the outer side of the membrane. Concurrently, at the inner side of the membrane, the CO production rate initially drops because of recombination with the released O_2 and then increases and levels off to twice the molar value of the O_2 production rate as steady-state is reached in the exchange of oxygen ions, electrons (Ce^{3+}/Ce^{4+} change), and vacancies. The mass balance is therefore closed. The differing timescales for the stabilization of CO and O_2 are attributed to the different inlet flow rates of Ar and CO_2 on either side of the membrane combined with the system time delay between the solar reactor exit and the gas measurement point downstream.

Scanning electron microscopy images of the surface and cross-section of the ceria membrane before and after 443 min of reaction indicate that the morphological structure is preserved (Figure S2). Further, X-ray diffraction and energy dispersive spectroscopy confirm that the bulk composition of the ceria membranes is unchanged and matches that of the pristine ceria powder used to manufacture the membranes (Figure S3). Figure 3 shows the X-ray photoelectron spectroscopy (XPS) spectra for the O 1s orbital of ceria at the inner and outer surfaces of the membrane before and after the reaction, as well as for the pristine ceria powder used to manufacture the membranes. The XPS signal is deconvoluted into three peaks, attributed to lattice oxygen (O^{2-}) at a binding energy of 529 eV, surface oxygen

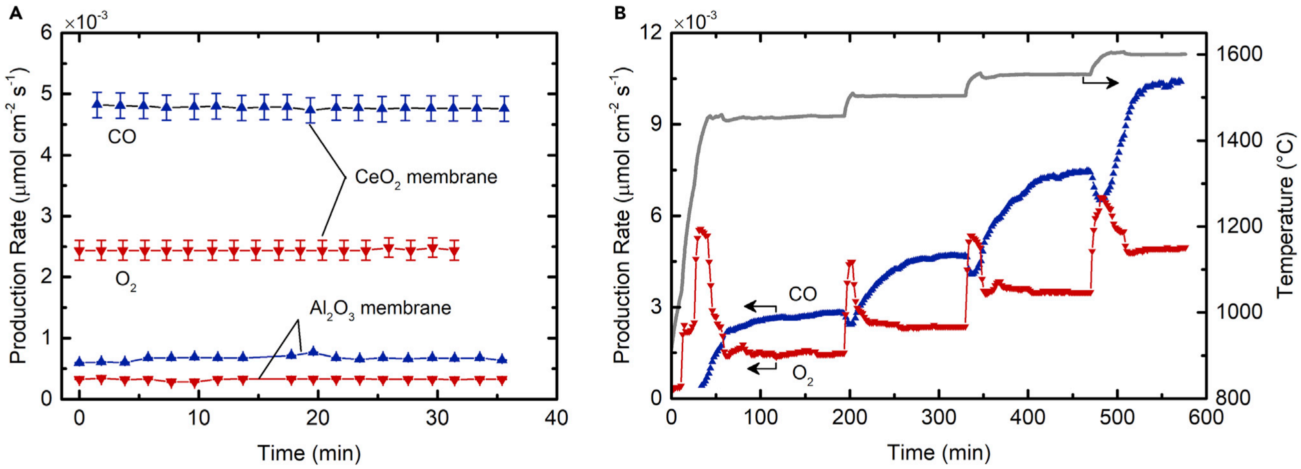

Figure 2. Transient and Steady-State Behavior of Specific Gas Production Rates

(A) Steady-state specific production rates of CO and O_2 during a CO_2-splitting run at 1,500°C with a redox-active CeO_2 membrane and a redox-inactive Al_2O_3 membrane. O_2 is measured on the outer side of the CeO_2 membrane and on the inner side of the Al_2O_3 membrane. Error bars are computed from device measurement uncertainties via error propagation (see Supplemental Information).

(B) Specific production rates of CO and O_2 with a CeO_2 membrane over the course of stepwise increase in the temperature from 1,450°C to 1,600°C. Experimental conditions: 3,000–3,500 suns radiative flux, 25 mL min^{-1} CO_2 (inner side), 200 mL min^{-1} Ar (outer side), 6×10^{-5} bar p_{O_2}, and ambient total pressure.

in the form of adsorbed −OH or H_2O at a binding energy of ≈531 eV, and lattice oxygen in an oxygen-deficient environment with O^{2-} vacancies at a binding energy of ≈532 eV.[22–24] The pristine ceria powder contains a mix of all three oxygen types. The membrane before reaction exhibits slightly higher O 1s binding energies compared to the ceria powder, indicative of O^{2-} vacancies formed during sintering, which varies spatially (Figures 3B and 3C) due to temperature gradients in the sintering process. After 443 min reaction at T = 1,450°C–1,550°C and $p_{O_2} = 6 \times 10^{-5}$ bar, the binding energy is clearly shifted to lower values than those before reaction, indicative of the net consumption of O^{2-} vacancies due to the CO_2 splitting reaction. Note that the difference in the peak intensity for the lattice oxygen is significantly higher than that caused by spatial variation in the O^{2-} vacancy concentration discussed above. The complete XPS analysis including the Ce 3d and C 1s spectra is provided in the Supplemental Information (Figures S4 and S5).

Figure 4 shows the measured steady-state gas production rates for CO and O_2 as a function of: (a) temperature in the range 1,450°C–1,600°C at $p_{O_2} = 6 \times 10^{-5}$ bar; and (b) p_{O_2} in the range 1×10^{-6} to 2×10^{-4} bar at T = 1,600°C. Also indicated are the thermodynamic limits (derived in the Supplemental Information, Figure S6). The steady-state gas production rates are proportional to $\exp(-E_A/RT)$, where the apparent activation energy $E_A = 228 \pm 9$ kJ mol^{-1} CO matches the reaction enthalpy of 279 kJ mol^{-1} in this temperature range, suggesting conditions approaching thermodynamic equilibrium. The gas production rates decrease with p_{O_2} as expected from the CO_2-thermolysis equilibrium given by $K(T) = p_{CO} \cdot p_{O_2}^{1/2}/p_{CO_2}$ (Supplemental Information). However, particularly at $p_{O_2} < 5 \times 10^{-5}$ bar, the thermodynamic limit is not approached. Interestingly, the specific CO and O_2 production rates per unit area of membrane increase with the volumetric CO_2 flow rate (Figure S7). This dependency presumably arises from mass transfer limitations, as the relatively low CO_2 flow rate is unable to sweep away the CO product effectively on the inner side of the membrane. While the very high temperature and, in turn, relatively high activity of reactive oxygen vacancies at the inner surface

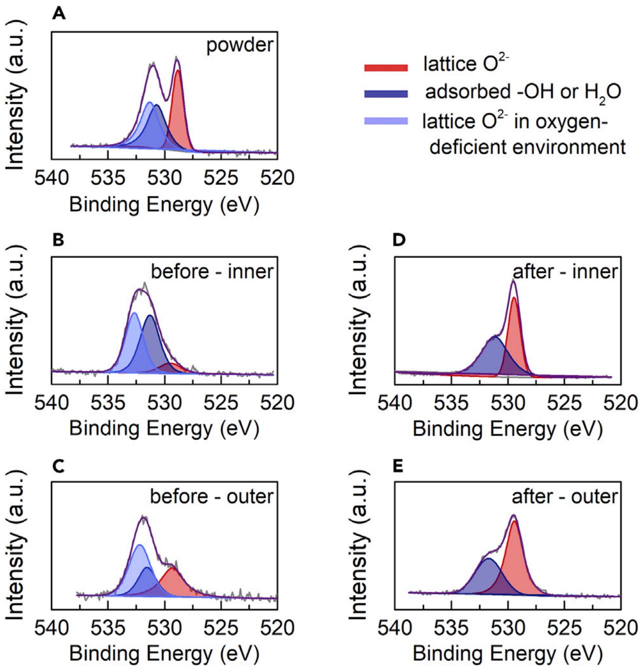

Figure 3. O 1s XPS Spectra between 540 and 525 eV

(A–E) Pristine ceria powder (A), the ceria membrane inner surface analyzed before (B), the ceria membrane outer surface analyzed before (C), the ceria membrane inner surface analyzed after (D), and the ceria outer surface analyzed after (E) a CO_2 splitting run of 443 min at 1,450°C–1,550°C and 6×10^{-5} bar O_2. The XPS deconvolution peaks are attributed to the lattice oxygen (red), surface oxygen in the form of adsorbed –OH or H_2O (dark blue), and lattice oxygen in oxygen-deficient regions (light blue), listed from lowest to highest binding energy. See also Figures S4 and S5.

of the membrane are expected to control the kinetics of the surface exchange reaction transferring oxygen from the gas phase into the solid phase, steady-state gas production rates may however be limited as well by this surface exchange. Thus, as anticipated,[4] the overall kinetics were not controlled by solid-state diffusion within the crystal lattice of ceria, consistent with previous observations with a solar cavity-receiver containing a porous structure directly exposed to high-flux irradiation.[25] This was indeed expected from the measured values of ambipolar diffusion coefficients of oxygen in ceria in the range 1.5×10^{-5}–4×10^{-4} cm^2 s^{-1} for 1,400°C–1,550°C,[4] which translated to reduction times in the order of seconds for the length scales across the 0.5 mm-thick membrane. Thus, as far as solid-state diffusion is concerned, the transport of oxygen vacancies through the membrane is almost instantaneous compared with the timescales of Figure 2B.

One key metric for the solar reactor performance is the solar-to-fuel energy conversion efficiency, defined as the ratio of the heating value of the fuel produced to the solar energy input. The theoretical limit exceeds 40% at 1,600°C (see Thermodynamic Analysis in Supplemental Information), in agreement with a comparable thermodynamic study,[26] but the measured value in this proof-of-concept prototype was less than 1% because no attempt was made to optimize the design. Straight-forward measures to boost the efficiency include the incorporation of an array of multiple tubular membranes inside the cavity-receiver for increasing the reaction surface and the recovery of the sensible heat of the outlet gas streams for preheating the inlet gas streams.

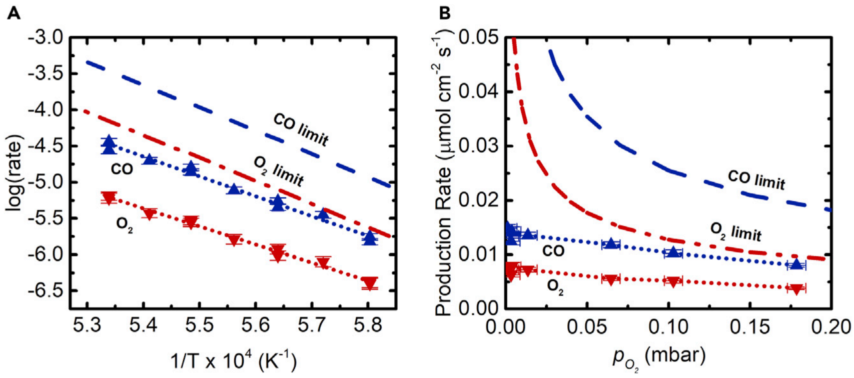

Figure 4. Thermodynamic Trends in Gas Production Rates

(A and B) Average steady-state gas production rates as a function of: (A) temperature in the range 1,450°C–1,600°C with uncertainty of ±0.6°C at $p_{O_2} = 6 \times 10^{-5}$; and (B) the partial pressure of O_2 at the Ar inlet in the range 1×10^{-6}–2×10^{-4} bar with uncertainty $\pm 6 \times 10^{-6}$ bar at T = 1,600°C. Dashed lines mark the thermodynamic limit. Dotted lines connect the data points for visual ease. See also Figure S7.

In sum, the feasibility of a solar membrane reactor for splitting CO_2 into separate streams of CO and O_2 has been demonstrated in terms of materials, selectivity, and stability. The design is simple, compact, modular, and scalable. The reaction rates were limited largely by mass and heat transfer, while the efficiency was limited mainly by the available membrane surface and the lack of heat recovery. Both limitations can be readily overcome through optimization and scale-up. Alternative membrane materials, e.g., doped ceria and perovskites, should exhibit rapid oxygen ion diffusivity at high operating temperatures while maintaining the thermochemical and structural stability of the membrane. Dual-membrane reactors for *in situ* removal of both O_2 and CO produced by CO_2-thermolysis should be explored for controlling the purity of the fuel produced. Notably, the large-scale implementation can be integrated to the concentrating solar tower and dish systems already established commercially. The solar production of CO from CO_2 can be coupled to the synthesis of liquid hydrocarbon fuels for sustainable transportation.

EXPERIMENTAL PROCEDURES

Materials

Cerium (IV) oxide (ceria, CeO_2, powder, particle size < 5 μm, 99.9% purity), poly (oxy-1,4-phenylene sulfonyl-1,4-phenylene) (PES, $(C_{12}H_8O_3S)_n$, pellets), polyvinylpyrrolidone (PVP, $(C_6H_9NO)_n$, powder, average M.W. 40,000), and 1-methyl-2-pyrrolidinone (NMP, C_5H_9NO, liquid, ≥ 99.0% purity) were from Sigma Aldrich. Coltogum high-temperature silicate adhesive (1,500°C maximum temperature) was from SFS unimarket AG. High-purity alumina adhesive (Resbond 989, Cotronics) was from Polytec PT GmbH. CO_2 (99.998%), Ar (99.996%, 99.999%), He (99.999%), and calibration gas mixtures, i.e., 500 mol ppm O_2 (99.999%) in Ar (99.999%), 500 mol ppm CO (99.997%) in Ar (99.999%), and 1,000 mol ppm CO (99.997%), 500 mol ppm CO_2 (99.995%), 500 mol ppm N_2 (99.999%), and 100 mol ppm O_2 (99.999%) in Ar (99.999%) were from Messer Schweiz AG. According to the manufacturer, Ar (99.996%) contained <5 ppm O_2 on volume basis, equivalent to a limiting $p_{O_2} < 5 \times 10^{-6}$ bar.

Membrane Fabrication

Capped tubular ceramic membranes were fabricated using a phase-inversion/sintering method.[27,28] PES (5.6–6.6 wt%) and PVP (0.4–0.5 wt%) were dissolved in NMP

(22.7–26.6 wt%) by stirring magnetically at 30°C. Ceria powder (66.4–71.2 wt%) was gradually suspended in the polymer solution, and the slurry was stirred in a covered beaker for at least 24 hr. The slurry was coated onto a membrane template made of silicone tubing (High-Flexible tubing, 3 mm ID, 7 mm OD, RCT Reichelt Chemietechnik GMbH & Co.), and the assembly was placed in a water bath for phase inversion (unfiltered tap water coagulant). The membrane precursor was then dried in air at 20°C and sintered for 8 hr at 1,600°C (oven model HT 64/17, Nabertherm). The sintered membranes were 5–6 mm OD, 4–5 mm in inner diameter (ID), and 150–250 mm in length. The typical wall thickness was less than 0.5 mm. In addition to the redox-active ceria membranes produced in house, commercial redox-inactive Al_2O_3 membranes (Alsint 99.7, 7 mm OD, 5 mm ID, 250 mm length, Intertechno-Firag AG) were used.

Solid-State Analysis

X-ray diffraction (XRD) was performed in the Bragg Brentano geometry using Cu Kα radiation (5-90° 2θ, 0.01° min^{-1} scan rate, 40 kV/40 mA output, Bruker D8 Advance). Basic structural data were obtained by multiphase Rietveld analysis (X'Pert HighScore). Scanning electron microscopy (SEM; 3 kV accelerating voltage, Hitachi, TM-1000) was used to analyze the membrane morphology. X-ray photoelectron spectroscopy (XPS) analyses were performed on a Phoibos 150 spectrometer calibrated at 84 eV against a clean Au(111) orientated single crystal. These analyses were carried out using a monochromatized Al Kα X-ray source with a photon energy of 1,486.7 eV and an energy resolution of about 0.8 meV. The sample area intercepted by X-rays was about 1.5 mm × 1.5 mm, and total pressures of the load lock and analysis chamber were 10^{-8} and 10^{-11} mbar, respectively. Survey (wide-scan) spectra and multiplex (narrow-scan) spectra of Ce 3d, O 1s, and C 1s were collected. The quantification and simulation of the experimental photo peaks were carried out using UNIFIT201. The line shapes used for curve fitting were a mix of Gaussian and Lorentian, and the background was fitted with the Shirley function.

Solar Experimental Setup

The tubular ceria membrane was placed inside a larger coaxial Al_2O_3 tube (Alsint 99.7, OD 25 mm, ID 20 mm, MTC Haldenwanger) through which Ar sweep gas was fed in an annular flow. A smaller coaxial Al_2O_3 tube (Rubalit, OD 3 mm, ID 1.5 mm, CeramTec) was placed inside the tubular membrane near the closed end to feed CO_2 in a countercurrent flow through the inner side of the membrane. This assembly was placed vertically inside a thermally insulated solar cavity-receiver with a 4 cm-diameter aperture. A compound parabolic concentrator of acceptance angle 45° was incorporated onto the aperture to boost the solar flux concentration by a factor of 2 and generate a more uniform directional distribution of concentrated radiation entering the cavity.[29] Experimentation was performed at the High-Flux Solar Simulator (HFSS) of ETH Zurich: an array of seven Xe-arcs, close-coupled to truncated ellipsoidal reflectors, provided an external source of intense thermal radiation that closely approximated the heat transfer characteristics of highly concentrating solar systems. The radiative flux distribution at the focal plane was measured optically using a calibrated charge-coupled device camera focused on a Lambertian (diffusely reflecting) target. The solar radiative power input through the aperture was calculated by integration of the radiative flux over the aperture area and verified with a water calorimeter. Temperatures were measured at the outer surface of the reactor shell using B-type thermocouples. Ar and CO_2 flow rates were regulated by electronic mass flow controllers (Bronkhorst F-201 C). Product gas composition was monitored on-line by gas chromatography (GC, Agilent 490 MicroGC).

Experimental Runs

All volume flow rates are given at standard conditions (1 bar and 0°C). During a typical run, the reactor was heated by the HFSS with a radiative power input of 2.0–2.5 kW to the desired nominal temperature in the range 1,300°C–1,600°C. The CO_2-splitting reaction was initiated by purging the membrane with 25 mL min^{-1} CO_2 (inside, oxidation side) and 100–800 mL min^{-1} Ar (outside, reduction side). The value of p_{O_2} in the Ar inlet flow included a small amount of air leakage into the system, as verified from the measured partial pressures of N_2, and could be controlled by varying the Ar flow rate. The compositions of both gas streams exiting the solar reactor were analyzed simultaneously using two GCs. Steady-state was defined here as the condition at which the measured gas concentration was within 2% of the mean over the previous five consecutive measurements collected at a frequency of one every 2 min:

$$\frac{\left| c_i(t_n) - \frac{1}{5}\sum_{j=n-5}^{n-1} c_i(t_j) \right|}{\frac{1}{5}\sum_{j=n-5}^{n-1} c_i(t_j)} \le 0.02 \qquad \text{(Equation 3)}$$

where $c_i(t_j)$ is the concentration of species i at time point j. Steady-state data were collected for at least 12 min, and the arithmetic mean was used to summarize the results at each experimental condition.

SUPPLEMENTAL INFORMATION

Supplemental Information includes Supplemental Text, seven figures, and one table and can be found with this article online at http://dx.doi.org/10.1016/j.joule.2017.07.015.

AUTHOR CONTRIBUTIONS

M.T., R.M., and A.S. designed the solar reactor. M.T. executed the experiments. R.M. and A.S. supervised the project.

ACKNOWLEDGMENTS

This work was funded in part by the Swiss Federal Office of Energy (No. SI/501213-01), the Swiss National Science Foundation (Ambizione Energy Grant No. 166883), and the European Research Council under the European Union's ERC Advanced Grant (SUNFUELS – No. 320541). We thank Michael Welte, Roberto Castiglioni, Raphael Röthlisberger, and Milos Baljozovic for supporting the experimental campaign.

Received: April 26, 2017
Revised: June 12, 2017
Accepted: July 27, 2017
Published: August 9, 2017

REFERENCES

1. Lewis, N.S., and Nocera, D.G. (2006). Powering the planet: chemical challenges in solar energy utilization. Proc. Natl. Acad. Sci. USA *103*, 15729–15735.
2. Ozin, G.A. (2015). Throwing new light on the reduction of CO2. Adv. Mater. *27*, 1957–1963.
3. Romero, M., and Steinfeld, A. (2012). Concentrating solar thermal power and thermochemical fuels. Energy Environ. Sci. *5*, 9234.
4. Ackermann, S., Scheffe, J.R., and Steinfeld, A. (2014). Diffusion of Oxygen in Ceria at Elevated Temperatures and Its Application to H2O/CO2Splitting Thermochemical Redox Cycles. J. Phys. Chem. C *118*, 5216–5225.
5. Mogensen, M., Sammes, N.M., and Tompsett, G.A. (2000). Physical, chemical and electrochemical properties of pure and doped ceria. Solid State Ion. *129*, 63–94.

6. Michalsky, R., Botu, V., Hargus, C.M., Peterson, A.A., and Steinfeld, A. (2015). Design Principles for Metal Oxide Redox Materials for Solar-Driven Isothermal Fuel Production. Adv. Energy Mater. *5*, 1401082.

7. Panlener, R.J., Blumenthal, R.N., and Garnier, J.E. (1975). A thermodynamic study of nonstoichiometric cerium dioxide. J. Phys. Chem. Solids *36*, 1213–1222.

8. Chueh, W.C., Falter, C., Abbott, M., Scipio, D., Furler, P., Haile, S.M., and Steinfeld, A. (2010). High-flux solar-driven thermochemical dissociation of CO2 and H2O using nonstoichiometric ceria. Science *330*, 1797–1801.

9. Muhich, C.L., Evanko, B.W., Weston, K.C., Lichty, P., Liang, X., Martinek, J., Musgrave, C.B., and Weimer, A.W. (2013). Efficient generation of H2 by splitting water with an isothermal redox cycle. Science *341*, 540–542.

10. Hao, Y., Yang, C.K., and Haile, S.M. (2013). High-temperature isothermal chemical cycling for solar-driven fuel production. Phys. Chem. Chem. Phys. *15*, 17084–17092.

11. Venstrom, L.J., De Smith, R.M., Hao, Y., Haile, S.M., and Davidson, J.H. (2014). Efficient Splitting of CO2in an Isothermal Redox Cycle Based on Ceria. Energy Fuels *28*, 2732–2742.

12. Fletcher, E.A., and Moen, R.L. (1977). Hydrogen- and oxygen from water. Science *197*, 1050–1056.

13. Noring, J.E., Diver, R.B., and Fletcher, E.A. (1981). Hydrogen and oxygen from water - v. the ROC system. Energy *6*, 109–121.

14. Michalsky, R., Neuhaus, D., and Steinfeld, A. (2015). Carbon Dioxide Reforming of Methane using an Isothermal Redox Membrane Reactor. Energy Technol. (Weinheim) *3*, 784–789.

15. Evdou, A., Zaspalis, V., and Nalbandian, L. (2010). La1−xSrxFeO3−δ perovskites as redox materials for application in a membrane reactor for simultaneous production of pure hydrogen and synthesis gas. Fuel *89*, 1265–1273.

16. Jin, W., Zhang, C., Chang, X., Fan, Y., Xing, W., and Xu, N. (2008). Efficient catalytic decomposition of CO2 to CO and O2 over Pd/mixed-conducting oxide catalyst in an oxygen-permeable membrane reactor. Environ. Sci. Technol. *42*, 3064–3068.

17. Franca, R.V., Thursfield, A., and Metcalfe, I.S. (2012). La0.6Sr0.4Co0.2Fe0.8O3−δ microtubular membranes for hydrogen production from water splitting. J. Membr. Sci. *389*, 173–181.

18. Nalbandian, L., Evdou, A., and Zaspalis, V. (2009). La1−xSrxMO3 (M = Mn, Fe) perovskites as materials for thermochemical hydrogen production in conventional and membrane reactors. Int. J. Hydrogen Energy *34*, 7162–7172.

19. Jeon, S.-Y., Im, H.-N., Singh, B., Hwang, J.-H., and Song, S.-J. (2013). A thermodynamically stable La2NiO4+δ/ Gd0.1Ce0.9O1.95 bilayer oxygen transport membrane in membrane-assisted water splitting for hydrogen production. Ceram. Int. *39*, 3893–3899.

20. Wu, X.Y., Chang, L., Uddi, M., Kirchen, P., and Ghoniem, A.F. (2015). Toward enhanced hydrogen generation from water using oxygen permeating LCF membranes. Phys. Chem. Chem. Phys. *17*, 10093–10107.

21. Balachandran, U., Lee, T.H., and Dorris, S.E. (2007). Hydrogen production by water dissociation using mixed conducting dense ceramic membranes. Int. J. Hydrogen Energy *32*, 451–456.

22. Yang, Y., Jin, Y., He, H., and Ye, Z. (2010). Facile synthesis and characterization of ultrathin cerium oxide nanorods. CrystEngComm *12*, *2663*.

23. Younis, A., Chu, D., and Li, S. (2012). Oxygen level: the dominant of resistive switching characteristics in cerium oxide thin films. J. Phys. D Appl. Phys. *45*, 355101.

24. Zhang, J., Zhao, H., Wei, F., Yang, M., Yang, Z., Chen, Q., and Chen, J. (2014). Resistive switching behaviour of highly epitaxial CeO_2 thin film for memory application. Rapid Research Letters *8*, 95–99.

25. Furler, P., Scheffe, J., Gorbar, M., Moes, L., Vogt, U., and Steinfeld, A. (2012). Solar thermochemical CO_2 splitting utilizing a reticulated porous ceria redox system. Energy Fuels *26*, 7051–7059.

26. Zhu, L., Lu, Y., and Shen, S. (2016). Solar fuel production at high temperatures using ceria as a dense membrane. Energy *104*, 53–63.

27. He, W., Huang, H., Gao, J.-f., Winnubst, L., and Chen, C.-s. (2014). Phase-inversion tape casting and oxygen permeation properties of supported ceramic membranes. J. Membr. Sci. *452*, 294–299.

28. Tan, X., Liu, Y., and Li, K. (2005). Preparation of LSCF Ceramic Hollow-Fiber Membranes for Oxygen Production by a Phase-Inversion/Sintering Technique. Ind. Eng. Chem. Res. *44*, 61–66.

29. Welford, W.T., and Winston, R. (1989). High Collection Nonimaging Optics (San Diego: Academic Press, Inc.).

JOUL, Volume *1*

Supplemental Information

Solar-Driven Thermochemical Splitting of CO_2 and *In Situ* Separation of CO and O_2 across a Ceria Redox Membrane Reactor

Maria Tou, Ronald Michalsky, and Aldo Steinfeld

Supplemental Text

Reference experiments of direct thermolysis with a redox-inactive Al_2O_3 membrane were conducted at 1300, 1400, and 1500°C. No O_2 production was detected on the outer (reduction) side. In the inner (oxidation) side, the average O_2:CO molar ratio was 0.49 ± 0.03, indicating a closed mass balance. The measured CO_2 conversion matched the magnitude expected for equilibrium thermolysis (see section Thermodynamic Analysis). Small discrepancies arose presumably from non-uniform temperatures in the reaction zone as well as O_2/CO partial recombination upon cooling the product gas to ambient temperature. The longest CO_2-splitting experiment conducted in this study maintained steady-state for 260 minutes, as shown in Fig. S1. Once steady state was reached at 1475°C, the production rates of CO and O_2 remained constant, thus indicating stable reactor performance. The O_2:CO ratio was 0.52 ± 0.05.

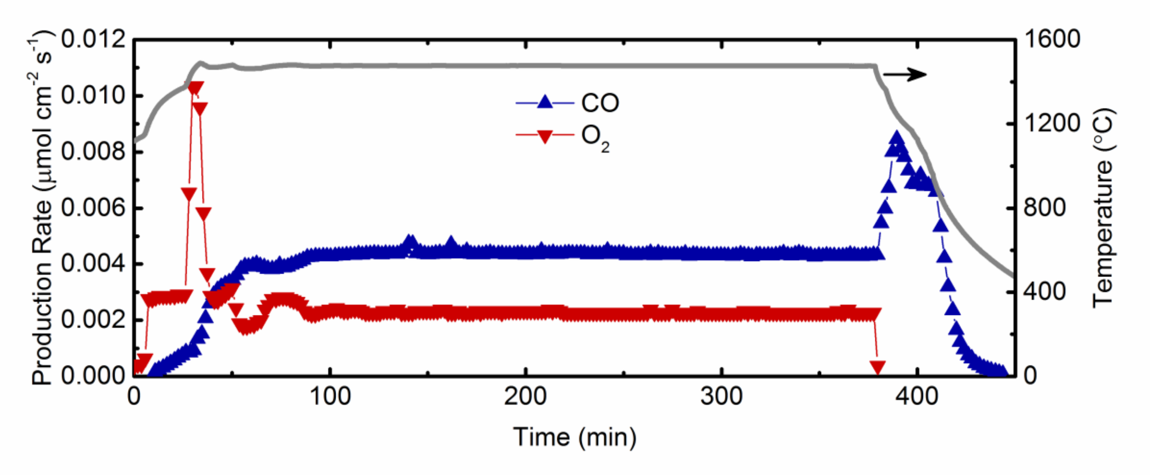

Fig. S1. Solar experimental run for CO_2 splitting with a ceria redox membrane.

<u>Materials characterization</u>

Figures S2a and S2b show SEMs of the membrane surface before and after a CO_2 splitting run, respectively. The dense surface exhibits some defects visible as dark spots on or between ceria grains, attributed to the pore structure of grains that have not fully sintered. Contamination was considered negligible as verified by energy dispersive spectroscopy (EDS). In general, pores do not pose a priori a problem provided they are unconnected and do not extend through the entire cross-section. This characteristic is necessary to keep the oxidizing and reducing streams separate and is seen in the membrane cross-section in Figs. S2c and S2d before and after a CO_2 splitting run, respectively. Mass transfer across the membrane is exclusively by diffusion of oxygen ions (O^{2-}) through the crystal lattice. The inner and outer surfaces of the tubular membrane can be distinguished in the SEM images by the curvature of the cross-section. The larger pores on the outer surface can be attributed to the phase-inversion synthesis technique and the inward shrinkage during sintering. Membrane thickness is typically less than 0.5 mm, which is below the critical length where O^{2-} diffusion in ceria becomes rate limiting.[4]

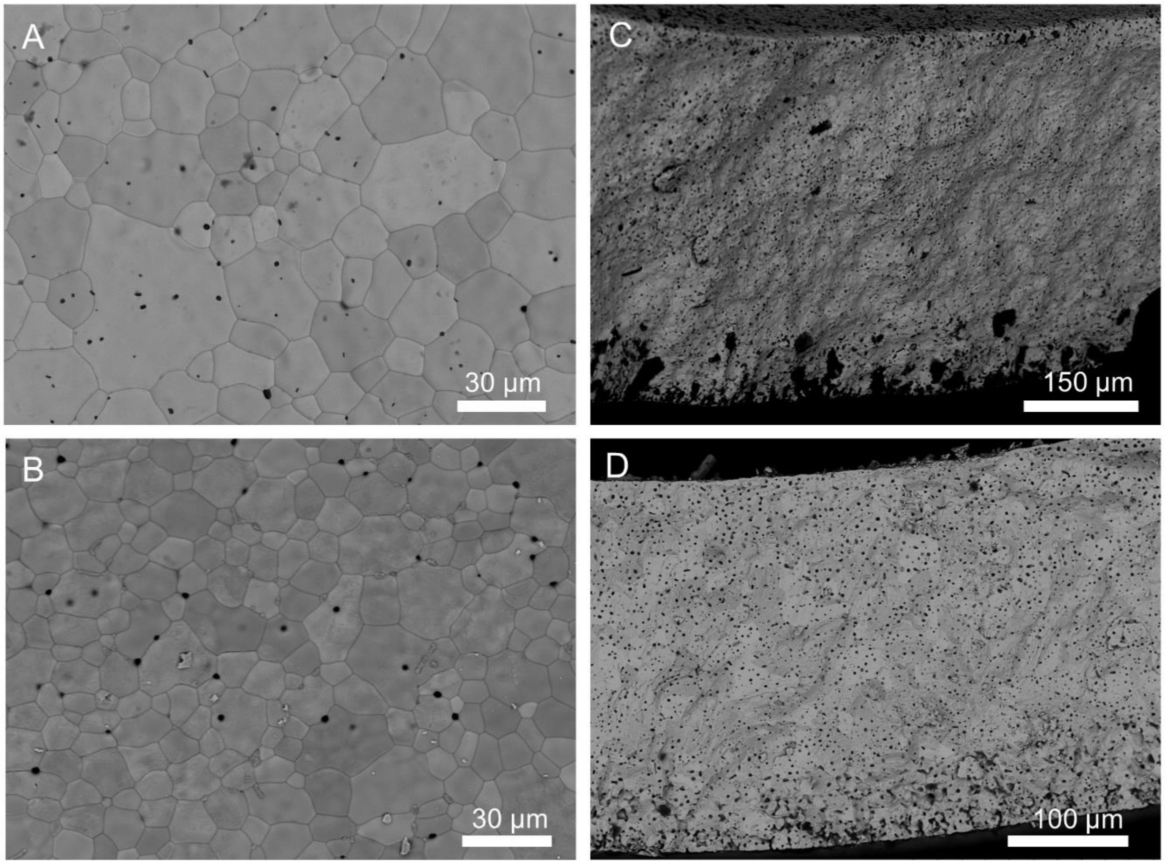

Fig. S2. SEM images of the ceria membrane surface (a, b) and cross section (c, d) analyzed before (a, c) and after (b, d) a CO_2 splitting run in the solar reactor.

Figure S3 shows the XRD spectra of pristine (as-purchased) ceria powder (i), and of the ceria membrane before (ii) and after (iii) a CO_2 splitting run in the solar reactor. The membrane composition is unchanged before and after reaction and matches the pristine ceria powder. Diffraction peaks are narrower in the spectra corresponding to membranes than in those from the powder due to the sintering of particles during fabrication.

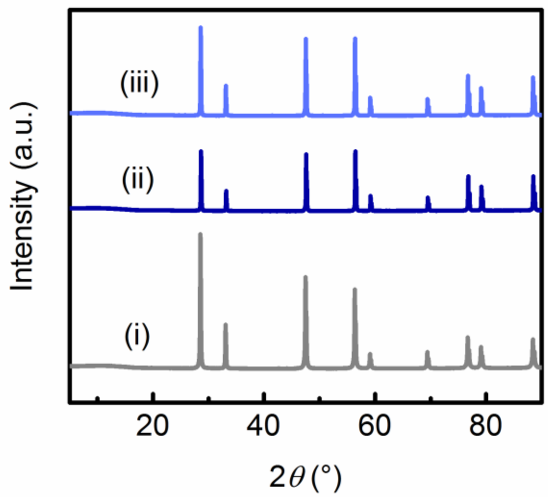

Fig. S3. XRD spectra of pristine ceria powder (i), and of the ceria membrane before (ii) and after (iii) a CO_2 splitting run in the solar reactor.

Figure S4 shows the XPS spectra for the C 1s orbital of ceria at the inner and outer surfaces of the membrane before and after reaction, as well as for the pristine ceria powder used to manufacture the membranes. The primary peak at ~285 eV is adventitious carbon attributed to adsorbed carbonaceous species from air.[30,31] Peaks appearing at ~286 eV and at ~288 eV are attributed to oxidized carbonaceous species also arising from exposure to air, where the higher binding energy peak is associated with carbonates.[30-32] Carbonaceous species were detected in all samples, which was expected as samples were stored in air at ambient conditions, without any cleaning before analysis.

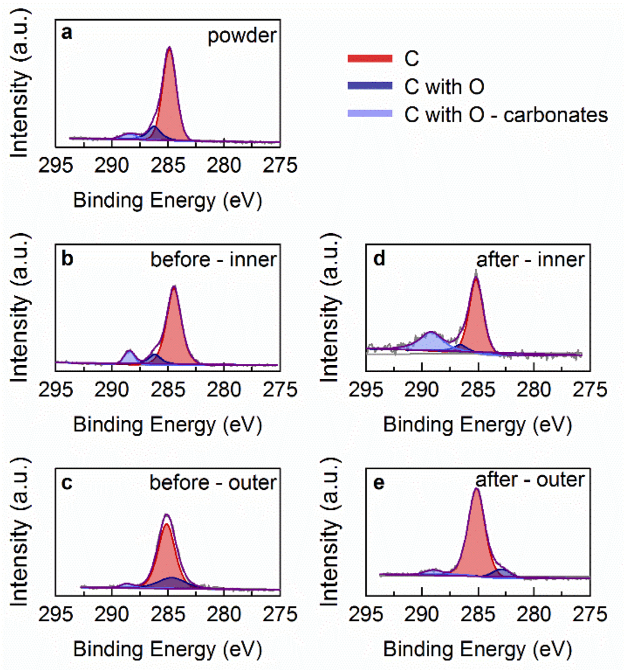

Fig. S4. C 1s XPS spectra between 295 and 275 eV of (a) $CeO_{2-\delta}$ powder as-purchased, and of the $CeO_{2-\delta}$ membrane (b, d) outer surface, and (c, e) inner surface, analyzed (b, c) before and (d, e) after CO_2 splitting for 443 min at 1450-1550°C and 6×10^{-5} bar O_2. The XPS deconvolution peaks are attributed to adventitious carbon (red) and oxidized carbon species (dark blue, light blue), where the higher binding energy peak is associated with carbonates (light blue).

Fig. S5 shows XPS spectra for the Ce 3d orbital of ceria at the inner and outer surfaces of the membrane before and after reaction, as well as for the pristine ceria powder used to manufacture the membranes. The XPS signal is deconvoluted into eight peaks, corresponding to four pairs of spin-orbit doublets, where peaks at ~897 and ~916 eV are attributed to Ce^{4+} and peaks at ~885 and ~903 eV are attributed to Ce^{3+}.[33-35] In general, the samples exhibited a mix of Ce^{4+} and Ce^{3+}, with Ce^{3+} as the relatively smaller fraction. The overall Ce 3d signal was small compared to O 1s across all samples.

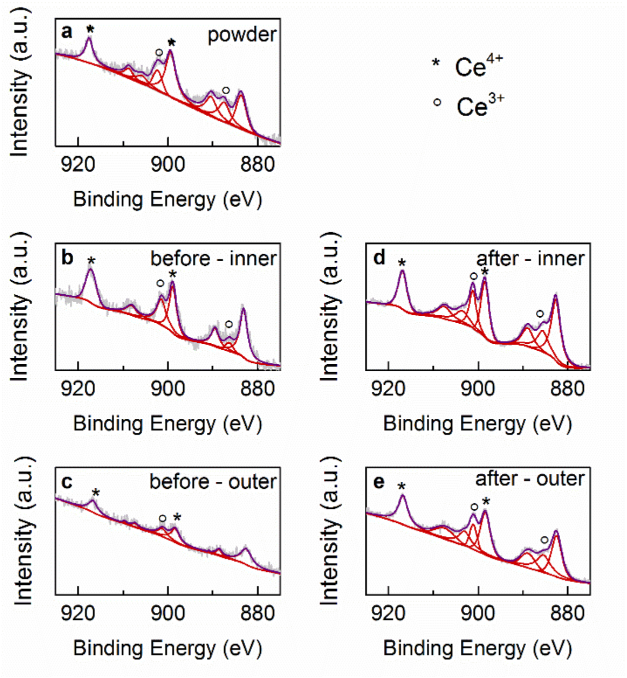

Fig. S5. Ce 3d XPS spectra between 925 and 875 eV of (a) $CeO_{2-\delta}$ powder as-purchased, and of the $CeO_{2-\delta}$ membrane (b, d) outer surface, and (c, e) inner surface, analyzed (b, c) before and (d, e) after CO_2 splitting for 443 min at 1450-1550°C and 6×10^{-5} bar O_2. Peaks attributed to Ce^{4+} and Ce^{3+} are denoted by asterisks (*) and open circles (°), respectively.

Thermodynamic Analysis

Assuming ideal gases for a reference state at 1 bar, the equilibrium constant of CO_2 thermolysis (CO_2 = CO + ½O_2) is given by

$$K(T) = \frac{p_{\mathrm{CO}}\sqrt{p_{\mathrm{O_2}}}}{p_{\mathrm{CO_2}}} = \exp\left(\frac{-\Delta G^{\circ}_{\mathrm{rxn}}}{RT}\right) \quad \text{(Eq. S1)}$$

where p_i is the partial pressure of gas species i, $\Delta G^{\circ}_{\mathrm{rxn}}$ is the standard Gibbs free energy change, and R is the universal gas constant. $K(T)$ was computed using thermodynamic data from NIST JANAF tables (http://kinetics.nist.gov/janaf/). Assuming a feed of pure CO_2, the gas composition must further satisfy $p_{\mathrm{total}} = p_{\mathrm{CO_2,i}} = p_{\mathrm{CO_2}} + p_{\mathrm{CO}} + p_{\mathrm{O_2}}$. The fundamental concept of the semi-permeable redox membrane is that it can shift the CO_2 thermolysis equilibrium by removing O_2 from the inner (oxidation) side and transporting it to the outer (reduction) side. Thus, the presence of the membrane introduces an additional degree of freedom besides T and p_{total}, namely setting $p_{\mathrm{O_2}}$ at the inlet on the outer (reduction) side of the membrane. This can be accomplished by vacuum pumping or, as in this case, by a purging flow of Ar. In the counter-current arrangement, $p_{\mathrm{O_2}}$ exiting the inner side equilibrates with $p_{\mathrm{O_2}}$ entering the outer side of the membrane and the product composition can be determined from Eq. S1. Thus, the driving force of CO_2 splitting can be expressed as the difference in $p_{\mathrm{O_2}}$ between the CO_2 inlet and outlet, the former dependent

on temperature only and the latter dependent on the p_{O_2} set in the reduction inlet. This Δp_{O_2} driving force is shown in Fig. S6a as a function of T and p_{O_2} at the reduction inlet in the ranges T = 1200-1700°C and p_{O_2} = 10^{-7}-10^{-3} bar at p_{total} = 1 bar. Evidently, Δp_{O_2} increases with temperature because of its correlation with the thermolysis equilibrium constant. Negative values of Δp_{O_2} indicate unfavorable conditions where the membrane provides no benefit. The conversion of CO_2 at equilibrium is shown in Fig. S6b at the same T and p_{O_2} conditions. As expected, the conversion increases monotonically with temperature and decreases with p_{O_2}. Unlike in a two-step redox cycle, a lower oxidation temperature detrimentally affects the thermolytic driving force.

The solar-to-fuel energy conversion efficiency, $\eta_{\text{solar-to-fuel}}$, is defined for steady-state CO_2 splitting as:

$$\eta_{\text{solar-to-fuel}} = \frac{\dot{n}_{\text{CO}} \cdot \text{HV}_{\text{CO}}}{\dot{Q}_{\text{solar}}} \quad \text{(Eq. S2)}$$

where $\dot{n}_{CO}$ is the molar production rate of CO, HV_{CO} is the heating value of CO (HV_{CO} = 283 kJ/mol) and $\dot{Q}_{solar}$ is the total solar radiative power input. $\dot{n}_{CO}$ is calculated from the equilibrium conversion of CO_2 at T, p_{O_2}, and p_{total} conditions (Fig. S6b). $\dot{Q}_{solar}$ accounts for the reaction enthalpy, sensible heating of feed gases, re-radiation losses through the aperture, heat losses through the walls, as well as the power required for the separation of product gases:

$$\dot{Q}_{\text{solar}} = \dot{Q}_{\text{rxn}} + \dot{Q}_{\text{sensible}} + \dot{Q}_{\text{reradiation}} + \dot{Q}_{\text{wall}} + \dot{Q}_{\text{separation}} \quad \text{(Eq. S3)}$$

The heat required for the reaction enthalpy is:

$$\dot{Q}_{\text{rxn}} = \dot{n}_{\text{CO}} \Delta H_{\text{rxn}}(T) \quad \text{(Eq. S4)}$$

The sensible heat term includes the power required to heat both the CO_2 and Ar gas streams from room temperature T_0 to the operating temperature T, less the heat recovered from the exiting hot gases with a heat exchanger efficacy η_{HEX}:[26,36-38]

$$\dot{Q}_{\text{sensible}} = \left[\dot{n}_{\text{CO}_2,i} \left(h_{\text{CO}_2}(T) - h_{\text{CO}_2}(T_0) \right) + \dot{n}_{\text{Ar},i} \left(h_{\text{Ar}}(T) - h_{\text{Ar}}(T_0) \right) \right] (1 - \eta_{\text{HEX}}) \quad \text{(Eq. S5)}$$

The change in the flow thermal properties due to the change in composition is neglected. The solar cavity-receiver is assumed to be a perfectly insulated blackbody absorber. Thus,[39]

$$\dot{Q}_{\text{reradiation}} = \left(\frac{\sigma T^4}{IC} \right) \dot{Q}_{\text{solar}} \quad \text{(Eq. S6)}$$

where I is the direct normal solar irradiation (DNI), C is the solar concentration ratio, and σ is the Stefan-Boltzmann constant. Heat loss through walls is proportional to the solar radiative power absorbed by the cavity, represented by a loss factor F,

$$\dot{Q}_{\text{wall}} = F \left(\dot{Q}_{\text{solar}} - \dot{Q}_{\text{reradiation}} \right) \quad \text{(Eq. S7)}$$

The energy required for product gas separation was computed from the minimum work based on the Gibbs free energy of mixing ideal gases in a binary mixture:

$$\dot{Q}_{\text{separation}} = \frac{RT}{\eta_{\text{Carnot}}} \left[\left(\dot{n}_{1,\text{f}} \ln x_{1,\text{f}} + \dot{n}_{2,\text{f}} \ln x_{2,\text{f}} \right) - \left(\dot{n}_{1,\text{i}} \ln x_{1,\text{i}} + \dot{n}_{2,\text{i}} \ln x_{2,\text{i}} \right) \right] \quad \text{(Eq. S8)}$$

where $x_{n,\text{i/f}}$ is the molar fraction of the nth component at the initial or final mixture composition. Carnot efficiency is assumed for the conversion of heat to work. The parametric values used in this analysis are summarized in Table S1. Fig. S6c shows $\eta_{\text{solar-to-fuel}}$ as a function of temperature in the range 1200-1700°C for p_{O_2} = 10^{-7}-10^{-3} bar and p_{total} = 1 bar. Evidently, $\eta_{\text{solar-to-fuel}}$ correlates with the CO_2 conversion. However, contrary to the trend in the CO_2 conversion, the incremental increase of $\eta_{\text{solar-to-fuel}}$ decreases with

increasing T and decreasing p_{O_2} levels. The results are in good agreement with previous studies making comparable assumptions.[26,36,37,40]

Table S1. Baseline parameters for the calculation of $\eta_{\text{solar-to-fuel}}$

Direct normal solar irradiation, I	1 kW m^{-2}
Solar concentration ratio, C	3000
Temperature, T	1500°C
Partial pressure of O_2 in Ar feed, p_{O_2}	10^{-5} bar
Total pressure, p_{total}	1 bar
Heat recovery efficacy, η_{HEX}	0.95
Ambient temperature, T_0	25°C
Heat loss factor, F	0.2
CO purity after separation	0.38

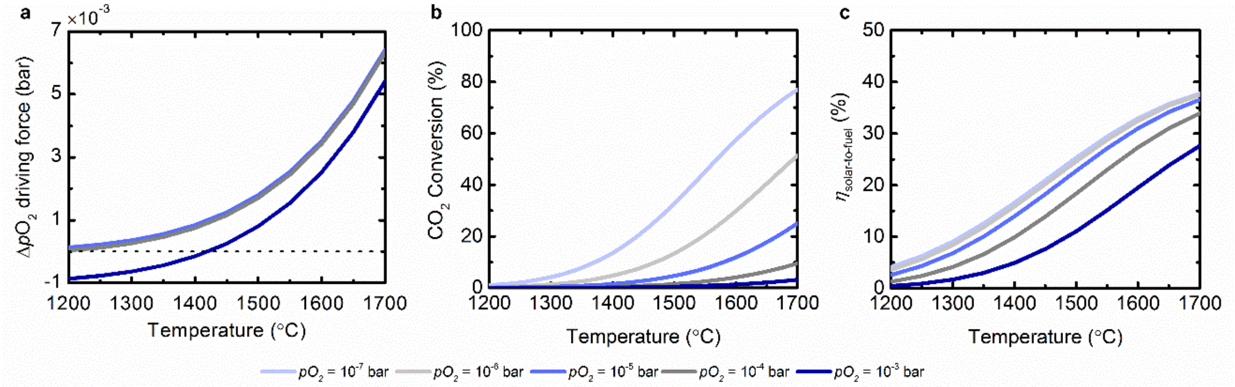

Fig. S6. For the conditions in the ranges T = 1200-1700°C and $p_{O_2} = 10^{-7}$-10^{-3} bar at p_{total} = 1 bar: (a) Δp_{O_2} driving force for CO_2 splitting; (b) CO_2 conversion at equilibrium; and (c) the solar-to-fuel energy conversion efficiency. The parameter is p_{O_2} at the Ar inlet on the reduction side of the redox membrane.

A sensitivity analysis on $\eta_{\text{solar-to-fuel}}$ was conducted to identify critical parameters by varying their value by ±20%. η_{HEX} had the largest effect: a 20% reduction resulted in $\Delta\eta_{\text{solar-to-fuel}}$ = -15% while perfect heat recovery led to $\Delta\eta_{\text{solar-to-fuel}}$ = +40% relative to the base case. Varying the other parameters changed $\eta_{\text{solar-to-fuel}}$ by less than 2%. To separate O_2 and recycle Ar, waste heat can be employed to drive a thermochemical redox cycle with perovskites.[41] Depending on the downstream use of CO, there may not be a need to separate it from unreacted CO_2, especially for high CO_2 conversions, as shown for syngas-to-liquid processing for the synthesis of kerosene[42] and methanol.[43]

Steady-state gas production rate limitations

Experimental CO_2 conversion rates deviated from the equilibrium values, especially at low p_{O_2}. The thermodynamic analysis assumes plug flow, setting an ideal upper limit for the gas production rates.

However, because of the boundary layer formation, the p_{O_2} near the walls may be greater than in the bulk, which could account in part for the experimental disparity from the calculated limit. We speculate that a possible cause for greater deviations at low p_{O_2} was mass transfer limitation on the inner (oxidation) side, because the constant CO_2 flow rate could not adequately remove CO from the reaction site. To check this hypothesis, an additional set of experiments were conducted at 1600°C with a fixed Ar flow rate and varied CO_2 flowrates. Fig. S7 shows the average steady-state gas production rates vs. p_{O_2} for various CO_2 flowrates. Dashed lines mark the thermodynamic limit; dotted lines connect the data points for visual ease. For clarity, the abscissa is plotted on a logarithm scale. This figure is a reproduction of the data of Fig. 4b, obtained for 25 mL min^{-1} CO_2, overlaid with hollow symbols representing the additional data points at 50, 100, and 200 mL min^{-1} CO_2. Higher CO_2 flowrates resulted in an increase in the production towards the thermodynamic limit, indicative of mass transfer limitation. However, similar results would be expected of a surface exchange reaction limited conversion. The maximum CO production rate across all experiments was 0.024 µmol s^{-1} per cm^2 of membrane, measured at T = 1600°C and p_{O_2} = 3×10^{-6} bar.

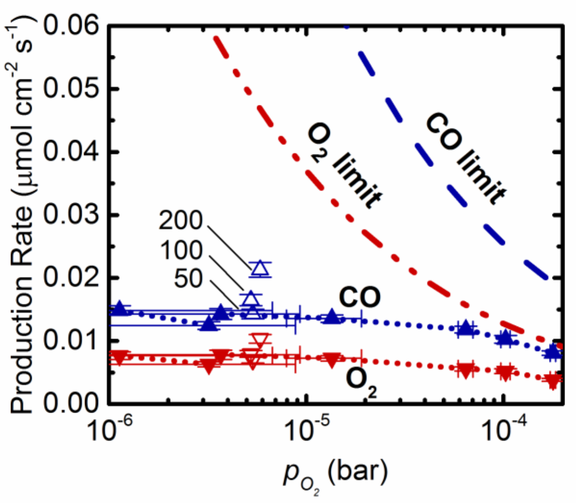

Fig. S7. Average steady-state gas production rates at 1600°C for various CO_2 flow rates versus the partial pressure of O_2 at the Ar inlet (in the range 1×10^{-6} - 2×10^{-4} bar with uncertainty ±6×10^{-6} bar). Error bars are computed from device measurement uncertainties via error propagation. Solid symbols represent data at 25 mL min^{-1} CO_2, originally shown in Fig. 4b. Dotted lines connect these data points for visual ease. Hollow symbols represent data points at 50, 100, and 200 mL min^{-1} CO_2, in order of increasing production rate. Dashed lines mark the thermodynamic limit.

Error analysis

The membrane was tested for gas-tightness by purging with CO_2 (inner side) and Ar (outer side) at ambient temperature. The CO_2 concentration in the outer side was below 10 ppm – the GC's detection limit. An O_2 leak rate of up to 0.015 µmol O_2 s^{-1} summed over both sides was derived from ambient air, which was taken into account in the measurement of the O_2 production rate. To distinguish the O_2 derived from CO_2, the small amount of ambient O_2 entering the system was calculated from the measured partial pressure of N_2. No CO was ever measured on the outer (reduction) side, even at high temperatures. The specific molar production rates of CO and O_2 were calculated as:

$$\dot{n}_{\mathrm{CO}} = \frac{\rho_{\mathrm{CO}} \dot{v}_{\mathrm{CO_2}}}{A_{\mathrm{membrane}} M_{\mathrm{CO}}} x_{\mathrm{CO}} \quad \text{(Eq. S9)}$$

$$\dot{n}_{O_2} = \frac{\rho_{O_2}\dot{v}_{Ar}}{A_{membrane}M_{O_2}}x_{O_2} \quad \text{(Eq. S10)}$$

where $A_{membrane}$ is the active area of the membrane, x_i is the volume fraction of species i on the appropriate side measured by the GC, $\dot{v}_i$ is the standard volumetric flow rate of gas i, and ρ_i and M_i are the standard density and molar mass of species i. Error bars for $\dot{n}_{CO}$ and $\dot{n}_{O2}$ were calculated using error propagation analysis. The uncertainties were quantified for each measured value, namely: x_i, v_i, $A_{membrane}$. For the GC measurements, uncertainty was taken to be the precision limit of 10 ppm. The uncertainty in the mass flow controllers was reported by Bronkhorst as: $\delta\dot{v} = (0.5\%\mathrm{Rd}) + (0.1\%\mathrm{FS})$, where the first term refers to the percentage of the reading (Rd) and the second refers to the percentage of the full-scale (FS) capacity of the mass flow controller (Bronkhorst Datasheet F-201CV). The same error propagation approach was used to evaluate the uncertainty in the molar ratio O_2:CO,

$$\delta\left(\frac{\dot{n}_{O_2}}{\dot{n}_{CO}}\right) = \frac{\dot{n}_{O_2}}{\dot{n}_{CO}}\left[\left(\frac{\delta\dot{n}_{O_2}}{\dot{n}_{O_2}}\right)^2 + \left(\frac{\delta\dot{n}_{CO}}{\dot{n}_{CO}}\right)^2\right]^{1/2} \quad \text{(Eq. S11)}$$

Supplemental References

30 Barr, T.L., and Seal, S. (1995). Nature of the use of adventitious carbon as a binding energy standard. Journal of Vacuum Science & Technology A: Vacuum, Surfaces, and Films *13*, 1239-1246.

31 Batis, N.H., Delichere, P., and Batis, H. (2005). Physicochemical and catalytic properties in methane combustion of La1−xCaxMnO3±y (0≤x≤1; −0.04≤y≤0.24) perovskite-type oxide. Applied Catalysis A: General *282*, 173-180.

32 Moulder, J.F. (1992). Handbook of X-ray Photoelectron Spectroscopy: A Reference Book of Standard Spectra for Identification and Interpretation of XPS Data (Physical Electronics Division, Perkin-Elmer Corporation).

33 Xiao, W., Guo, Q., and Wang, E.G. (2003). Transformation of CeO2(1 1 1) to Ce2O3(0 0 0 1) films. Chemical Physics Letters *368*, 527-531.

34 Younis, A., Chu, D., and Li, S. (2012). Oxygen level: the dominant of resistive switching characteristics in cerium oxide thin films. Journal of Physics D: Applied Physics *45*, 355101.

35 Zhang, J., Zhao, H., Wei, F., Yang, M., Yang, Z., Chen, Q., and Chen, J. (2014). Resistive switching behaviour of highly epitaxial CeO2thin film for memory application. physica status solidi (RRL) - Rapid Research Letters *8*, 95-99.

36 Bader, R., Venstrom, L.J., Davidson, J.H., and Lipiński, W. (2013). Thermodynamic Analysis of Isothermal Redox Cycling of Ceria for Solar Fuel Production. Energy & Fuels *27*, 5533-5544.

37 Ermanoski, I., Miller, J.E., and Allendorf, M.D. (2014). Efficiency maximization in solar-thermochemical fuel production: challenging the concept of isothermal water splitting. Phys Chem Chem Phys *16*, 8418-8427.

38 Venstrom, L.J., De Smith, R.M., Hao, Y., Haile, S.M., and Davidson, J.H. (2014). Efficient Splitting of CO2in an Isothermal Redox Cycle Based on Ceria. Energy & Fuels *28*, 2732-2742.

39 Steinfeld, A., and Palumbo, R. (2001). Solar Thermochemical Process Technology. In Encyclopedia of Physical Science and Technology (Academic Press), pp. 237-256.

40 Wang, H., Hao, Y., and Kong, H. (2015). Thermodynamic study on solar thermochemical fuel production with oxygen permeation membrane reactors. International Journal of Energy Research *39*, 1790-1799.

41 Ezbiri, M., Allen, K.M., Galvez, M.E., Michalsky, R., and Steinfeld, A. (2015). Design Principles of Perovskites for Thermochemical Oxygen Separation. ChemSusChem *8*, 1966-1971.

42 Marxer, D., Furler, P., Scheffe, J., Geerlings, H., Falter, C., Batteiger, V., Sizmann, A., and Steinfeld, A. (2015). Demonstration of the Entire Production Chain to Renewable Kerosene via Solar Thermochemical Splitting of H2O and CO2. Energy & Fuels *29*, 3241-3250.

43 Ash-Kurlander, U., Martin, O., Fontana, L.D., Patil, V.R., Bernegger, M., Mondelli, C., Pérez-Ramírez, J., and Steinfeld, A. (2016). Impact of Daily Startup-Shutdown Conditions on the Production of Solar Methanol over a Commercial Cu-ZnO-Al2O3Catalyst. Energy Technology *4*, 565-572.
